# Lateral Root Initiation in Cucumber (*Cucumis sativus*): What Does the Expression Pattern of *Rapid Alkalinization Factor 34* (*RALF34*) Tell Us?

**DOI:** 10.3390/ijms24098440

**Published:** 2023-05-08

**Authors:** Alexey S. Kiryushkin, Elena L. Ilina, Elizaveta D. Guseva, Katharina Pawlowski, Kirill N. Demchenko

**Affiliations:** 1 Laboratory of Cellular and Molecular Mechanisms of Plant Development, Komarov Botanical Institute, Russian Academy of Sciences, 197022 Saint Petersburg, Russia; 2 Department of Ecology, Environment and Plant Sciences, Stockholm University, 10691 Stockholm, Sweden

**Keywords:** *Cucumis sativus* (cucumber), lateral root initiation, pericycle, plant peptide hormones, Rapid Alkalinization Factor 34, RALF34, root meristem, small signaling peptide, THESEUS1, xylem

## Abstract

In Arabidopsis, the small signaling peptide (peptide hormone) RALF34 is involved in the gene regulatory network of lateral root initiation. In this study, we aimed to understand the nature of the signals induced by RALF34 in the non-model plant cucumber (*Cucumis sativus*), where lateral root primordia are induced in the apical meristem of the parental root. The RALF family members of cucumber were identified using phylogenetic analysis. The sequence of events involved in the initiation and development of lateral root primordia in cucumber was examined in detail. To elucidate the role of the small signaling peptide *Cs*RALF34 and its receptor *Cs*THESEUS1 in the initial stages of lateral root formation in the parental root meristem in cucumber, we studied the expression patterns of both genes, as well as the localization and transport of the *Cs*RALF34 peptide. *CsRALF34* is expressed in all plant organs. *CsRALF34* seems to differ from *AtRALF34* in that its expression is not regulated by auxin. The expression of *AtRALF34,* as well as *CsRALF34,* is regulated in part by ethylene. *CsTHESEUS1* is expressed constitutively in cucumber root tissues. Our data suggest that *Cs*RALF34 acts in a non-cell-autonomous manner and is not involved in lateral root initiation in cucumber.

## 1. Introduction

Peptide hormones play a significant role in plant life by participating in the regulation of cell proliferation, chemical defense against herbivores and insects, the induction and development of nitrogen-fixing root nodules, the prevention of self-pollination, and other aspects of plant life [1,2,3].

The family of cysteine-rich Rapid Alkalinization Factors (RALF) peptides is ubiquitous in the plant kingdom; so far, 800 plant RALF peptides have been identified [4]. The mature peptide sequences can be grouped into four phylogenetically distinct clades [5]. In Arabidopsis, RALF peptides regulate root growth, cell elongation, cell wall integrity, plant immune signaling and other miscellaneous processes [4,6,7]. Some *RALF* genes show tissue-specific expression. For instance, Arabidopsis *RALF1*, *22*, *24*, *31*, *33*, *34* and *36* are expressed at different levels in the roots, leaves, and inflorescences [8,9,10,11]; *AtRALF4* is expressed in inflorescences, while the expression of *AtRALF19* and *AtRALF23* takes place in all organs [11]. The enumeration of the RALF family members was recently revised [12]; however, *At*RALF34 kept its previous position.

During lateral root initiation in Arabidopsis, *RALF34 (RALF-LIKE 34*, *RALFL34)* is expressed in the pericycle prior to the first asymmetric divisions [8]. At further stages of lateral root primordium formation, *AtRALF34* is expressed in both central and flanking pericycle cells. In addition, the activity of the *AtRALF34* promoter was visualized in the root cap and the rhizodermis [8,13]. Thieme et al. [14] demonstrated that *AtRALF34* mRNA could be transported from the root to the shoot. This means that the spatial profile of *AtRALF34* expression in the root is broader than that shown by Murphy et al. [8]. 

RALF peptides are produced as preproproteins and secreted into the apoplast, where some of them are activated by the proteolytic cleavage [15]. In tobacco, the RALF1 peptide was detected in the endoplasmic reticulum and in the apoplast, as shown by the fusion of a shortened peptide ORF with *GFP* [16]. The analysis of constructs containing the N-terminal signal sequence of *Solanum chacoense* RALF3 or the full-length ORF fused with the *GFP* sequence revealed the location of the peptide in the endoplasmic reticulum and in Golgi vesicles [17]. The *At*RALF34 preproprotein consists of 129 amino acid residues. After the cleavage of the N-terminal signal peptide during passage into the ER, the mature peptide has 56 amino acid residues and is predicted to be cleaved further by the Golgi-localized subtilase SITE-1 PROTEASE (S1P) (Olsson et al., 2019; Abarca et al., 2021). Similar to several other RALF peptides, *At*RALF34 contains four conserved cysteine residues that form disulfide bonds and are assumed to be important for its activity [18,19]. Like CLE peptides, RALF peptides act in a non-cell-autonomous manner [17,20].

The expression of *AtRALF34* appears to be regulated by APETALA2/ETHYLENE RESPONSE FACTORs (AP2/ERFs) from the ERF subfamily (ERF4, ERF9, ERF11, ERF105, and CYTOKININ RESPONSE FACTOR 3) [8]. The interactions of the *AtRALF34* promoter with ERF4 and ERF9 were analyzed further, leading to the conclusion that *RALF34* expression is negatively regulated by ERFs. The expression of *ERF4* and *ERF9* was upregulated by auxin, whereas *RALF34* expression was downregulated, consistent with the hypothesis that the expression of *RALF34* is regulated via the auxin-ERF4/ERF9 pathway. However, the role of ethylene in the regulation of *RALF34* expression through ERF4/ERF9 remains unclear. While the expression of *ERF4* and *ERF9* appeared to be sensitive to ethylene, *RALF34* expression was not affected by ethylene treatment [8].

The cell wall integrity-sensing receptor-like kinase (RLK) THESEUS1 (THE1) was identified as the pH-dependent receptor [21] or co-receptor [2,22] of *At*RALF34 (RALF34–THE1 module). Loss-of-function *the1* mutants showed a phenotype similar to that of *ralf34* mutants, displaying the increased density of lateral roots with the disruption of the first stage of lateral root development, which led to the irregular spacing of primordia and an increased proportion of stage I lateral root primordia, some of which were atypical in that they contained extra anticlinal cell divisions in the pericycle [21]. In addition, the RALF34–THE1 module is involved in the coordination of cell wall synthesis and cell elongation [23,24].

The Arabidopsis genome contains 17 members, forming the THESEUS1/FERONIA or *Catharanthus roseus* receptor-like kinase 1-like (*Cr*RLK1L) family [25]. *Cr*RLK1L proteins have a predicted intracellular serine-threonine kinase domain, which is highly conserved among all receptor-like kinases; a transmembrane domain [25]; and a variable extracellular domain including two malectin-like domains [26]. 

Additionally, for another member of the RALF family, *At*RALF1, a receptor from the *Cr*RLK1L family, FERONIA (FER), has been identified [27]. The autocrine signaling cascade triggered by the interaction of *At*RALF1 with the FER receptor promotes root hair growth and determines the final cell size [28]. It is likely that *At*RALF1–FER controls root growth suppression as a regulator of cell elongation [24] upon the inhibition of cellulose synthesis [22]; however, the specific developmental stages or environmental conditions in which the *At*RALF1–FER module is activated remain unclear [4]. 

In most flowering plants, the initiation of lateral root primordia takes place in the elongation zone of the parental root [29]. However, in some families, including Cucurbitaceae, an alternative mechanism of lateral root initiation is established directly in the apical meristem of the parental root [30,31,32]. Thus, root branching in cucumber (*Cucumis sativus*), an important crop with a well-studied genome, is initiated in the parental root meristem [33]. However, the earliest stages of this process are understudied, including the role of phytohormones. In particular, the signaling mechanisms underlying lateral root initiation in cucumber, including the role of peptide hormone signaling in the specification of founder cells, are poorly understood.

For instance, it remains an open question whether the RALF34–THE1 module also plays a role during lateral root initiation directly in the parental root meristem. This question can be addressed on the transcriptional level. Do the expression domains of the two genes match? How does the expression zone shift from the developing vascular tissues to the lateral root founder cells in the pericycle? Furthermore, is signal transmission resulting from the interaction of RALF34–THE1 involved in determining the competence of pericycle cells to initiate the formation of a lateral root meristem? Previous studies have shown that in Arabidopsis, RALF34 acts on the transcriptional cascade, leading to lateral root initiation at an earlier point than the GATA23 transcription factor [8], which until recently was considered the earliest molecular marker of lateral root founder cells [32,34]. 

In the present study, we identified *Cucumis sativus RALF34,* the ortholog of *Arabidopsis thaliana RALFL34*, based on sequence homology and phylogenetic analysis. We analyzed the expression levels of *CsRALF34* in different organs of cucumber plants, including seedling roots. Analysis of the effects of exogenously applied auxin, the precursor of ethylene, and inhibitor of ethylene biosynthesis, on *CsRALF34* expression showed no regulation by auxin and only partial regulation by ethylene. An examination of the expression of *CsRALF34* and its putative receptor *CsTHESEUS1*, as well as the localization of the *Cs*RALF34 peptide hormone, during the sequence of events involved in the initiation and development of lateral root primordia in cucumber, indicated that *Cs*RALF34 acts in a non-cell-autonomous manner and is not involved in lateral root initiation in cucumber.

## 2. Results

### 2.1. Identification of the Ortholog(s) of Arabidopsis Small Signaling Peptide RALF34 and Its Putative Receptor THESEUS1 in Cucumis sativus by Phylogenetic Analysis

**Figure 1:**
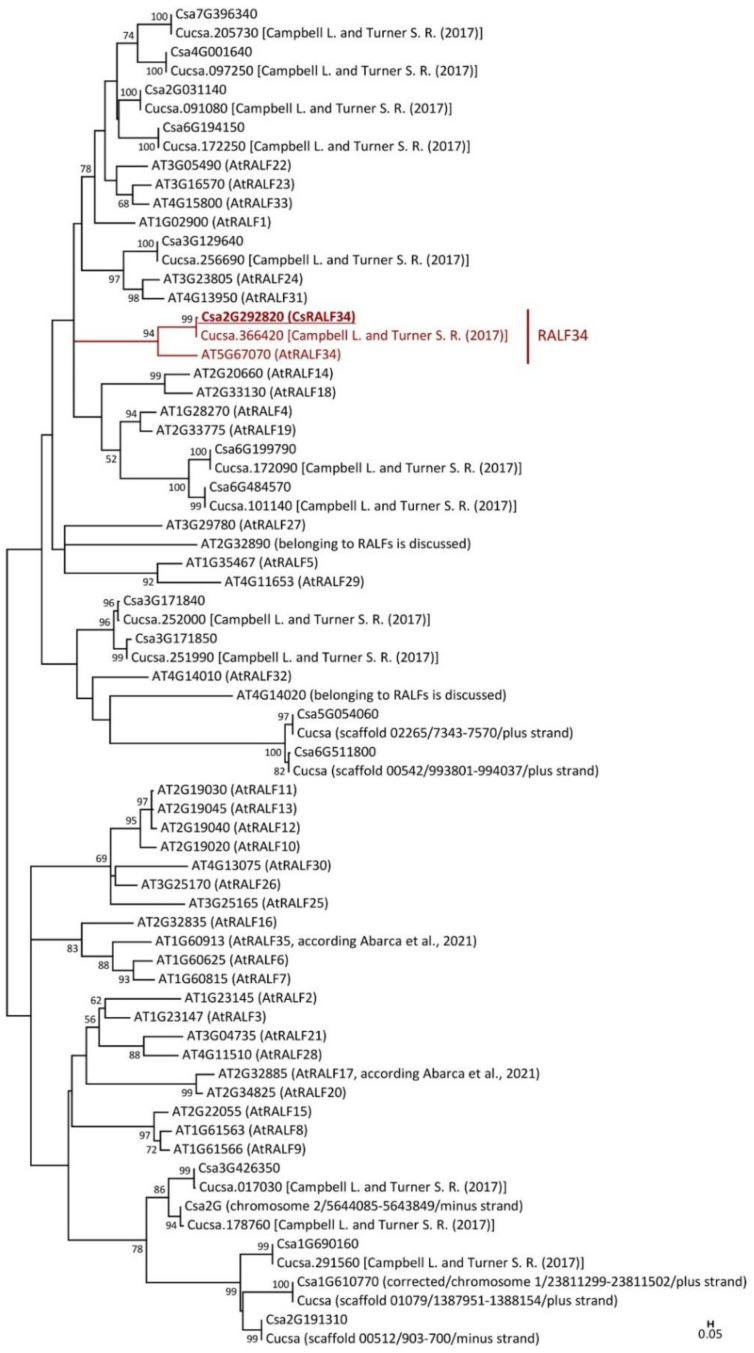
Phylogenetic tree of RALF proteins from *Arabidopsis thaliana* and *Cucumis sativus*. The putative *C. sativus* ortholog of AtRALF34 is indicated (red, underlined, bold). References are included for the *C. sativus* RALF proteins identified previously. A short annotation, including the number of the chromosome/scaffold and the positions of the RALF CDS and DNA strand, is provided for the five cucumber RALF peptides (Csa1G610770, Csa2G191310, Csa5G054060, Csa6G511800, and Cucsa.178760) encoded by genes not annotated in the genomes of *C. sativus* cv Gy14 v.1 and Chinese Long v.2, respectively. Gene ID prefixes: AT, *A. thaliana* based on the *Arabidopsis* Information Resource; Cucsa, *C. sativus* cv Gy14 v.1; and Csa, *C. sativus* cv Chinese Long v.2 based on Cucurbit Genomics Database v1. Scale bar: 0.05 amino acid substitutions per site. [5,12].

**Figure 2:**
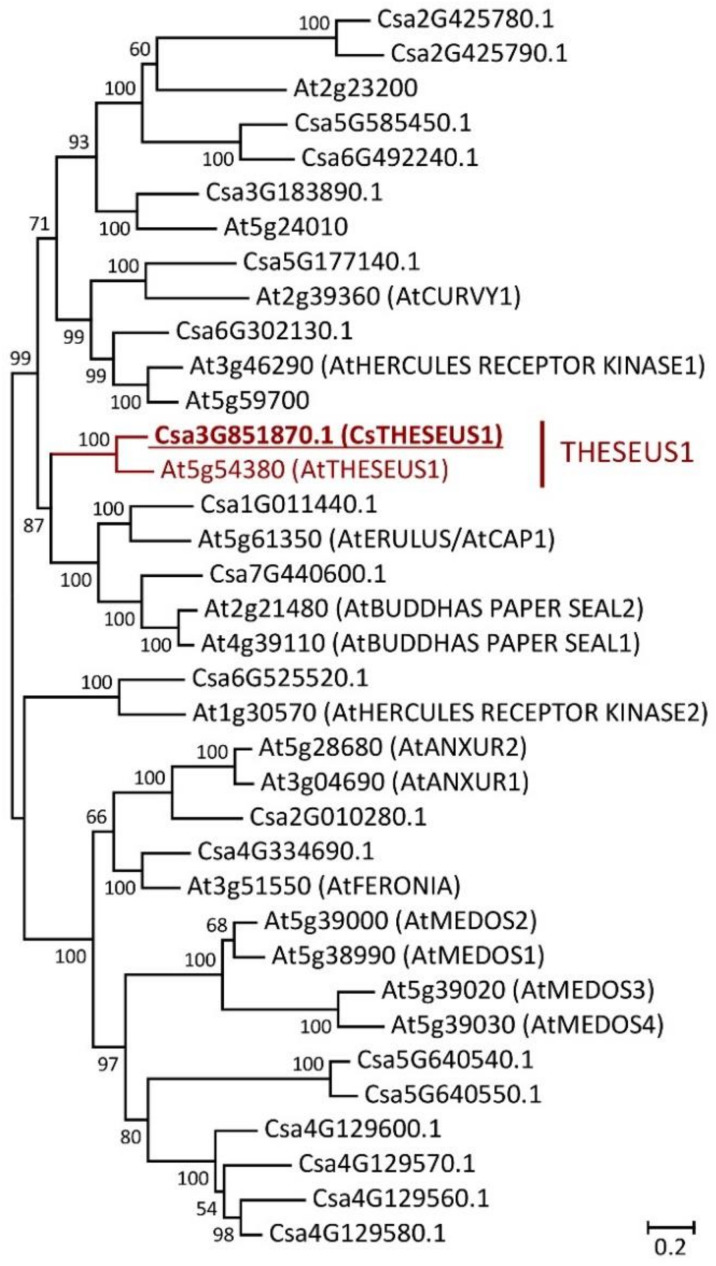
Phylogenetic tree of *Catharanthus roseus* RLK1–like receptor-like kinases (CrRLK1-like RLKs) from *Arabidopsis thaliana* and *Cucumis sativus*. The amino acid sequence of the putative ortholog of *At*THESEUS1 in *C. sativus* (indicated in red, underlined, bold) was analyzed together with all known sequences of CrRLK1-like RLKs from *A. thaliana* and *C. sativus*. Gene ID prefixes: At, *A. thaliana* on the *Arabidopsis* Information Resource; Csa, *C. sativus* Chinese Long v.2 on Cucurbit Genomics Database v1. Scale bar: 0.2 amino acid substitutions per site.

Thirty-seven Arabidopsis proteins were used for the search for RALF in the cucumber proteome, yielding 17 cucumber members of the RALF family (Figure 1). Of these, 13 had previously been identified by Campbell and Turner [5] in cucumber cultivar Gy14 v.1 (gene IDs from Cucurbit Genomics Database v1: Cucsa.017030; Cucsa.091080; Cucsa.097250; Cucsa.101140; Cucsa.172090; Cucsa.172250; Cucsa.178760; Cucsa.205730; Cucsa.251990; Cucsa.252000; Cucsa.256690; Cucsa.291560; Cucsa.366420), which were denoted on a phylogenetic tree (Figure 2). When the proteome of another cucumber cultivar (Chinese Long v.2) was analyzed, four additional RALF homologs were identified for the first time (gene IDs from Cucurbit Genomics Database v1: Csa1G610770; Csa2G191310; Csa5G054060; Csa6G511800). A comparison between the two cultivars revealed that some cucumber genes encoding RALF peptides were not annotated in cultivar Gy14 v.1, while they were annotated in cultivar Chinese Long v.2 (gene IDs: Csa1G610770; Csa2G191310; Csa5G054060; Csa6G511800). One gene from Gy14 v.1 (gene ID: Cucsa.178760) was not annotated in Chinese Long v.2 (Figure 1).

Phylogenetic analysis (Figure 1) showed that cucumber has a single ortholog of Arabidopsis RALF34 (*At*RALF34), which was named *Cs*RALF34 (gene IDs: Cucsa.366420/Csa2G292820). The number of cucumber and Arabidopsis RALF peptides differ, and the identification of orthologs is not always possible (Figure 1). Specifically, for 18 of the 37 Arabidopsis peptides (*At*RALF1, *At*RALF5–*At*RALF7, *At*RALF10–*At*RALF14, *At*RALF16, *At*RALF18, *AtRALF25*–*At*RALF27, *At*RALF29, *At*RALF30, and AT2G32890) we did not find any closely related cucumber RALF peptide amino acid sequences. However, the Cucsa.366420/Csa2G292820–*At*RALF34 branch represents a single sister pair within the Arabidopsis/cucumber RALF tree. Although cucumber has only 17 identified RALF peptides, we named Cucsa.366420/Csa2G292820 “*Cs*RALF34” (Figure 1), as was performed for another cucumber RALF, Csa6G484570, referred to as *Cs*RALF19 by Cheng et al. [35]. In cases of clear orthology, it is best to take over the Arabidopsis RALF numbering to avoid future nomenclatural confusion. The *Cs*RALF34 peptide showed 66% amino acid similarity and 54% amino acid identity with *At*RALF34 (Table S1). The OFR of *Cs*RALF34 was cloned and sequenced (Table S1).

Using BLASTN, TBLASTN and BLASTP, we also found single *At*RALF34 orthologs in 23 other Cucurbitales species (Figure S1, Table S1). The length of the RALF34 precursor varied across the analyzed Cucurbitales species from 115 to 132 amino acid residues; the peptides showed 62–70% amino acid similarity and 46–57% identity with the *At*RALF34 preproprotein (Table S1). At the same time, the number of amino acid residues of the Cucurbitales matures RALF34 peptides were the same as for *At*RALF34 (56 amino acids), and their subtilase cleavage sites were identical to that in *At*RALF34 as well (Figure S1, Table S1): the RALF34 preproproteins from all 24 Cucurbitales species examined contained the RRSL (Arg-Arg-Ser-Leu) sequence that is recognized by S1P subtilase (Figure S1, Table S1). The amino acid sequences of mature Cucurbitales RALF34 peptides showed 91–98% amino acid similarity and 79–84% identity with mature *At*RALF34 (Table S1).

The cucumber family of *Catharanthus roseus* RLK1–like receptor-like kinases (CrRLK1-like RLKs) consists of 19 proteins (Figure 2). One of these 19 proteins, Csa3G851870.1 (named *Cs*THESEUS1, *Cs*THE1), is a putative ortholog of the *At*THESEUS1 (*At*THE1) protein (Figure 2), the receptor of *At*RALF34 [21]. *Cs*THE1 has 73% amino acid similarity and 66% identity with *At*THE1. Apart from *Cs*THE1, we also identified several Arabidopsis CrRLK1-like RLKs that were sister-paired with cucumber CrRLK1-like RLKs. There were Csa3G183890.1, *Cs*CURVY1 (gene ID Csa5G177140.1), *Cs*ERULUS (gene ID Csa1G011440.1), *Cs*HERCULES RECEPTOR KINASE 2 (HERC2) (gene ID Csa6G525520.1), *Cs*FERONIA (gene ID Csa4G334690.1), representing putative orthologs of protein kinase At5g24010, *At*CURVY1 (gene ID At2g39360), *At*ERULUS (gene ID At5g61350), *At*HERC2 (gene ID At1g30570) and *At*FERONIA (gene ID At3g51550), respectively (Figure 2).

### 2.2. CsRALF34 Is Expressed Ubiquitously in Cucumber; Its Expression Is Not Affected by Auxin but Repressed by Ethylene

**Figure 3:**
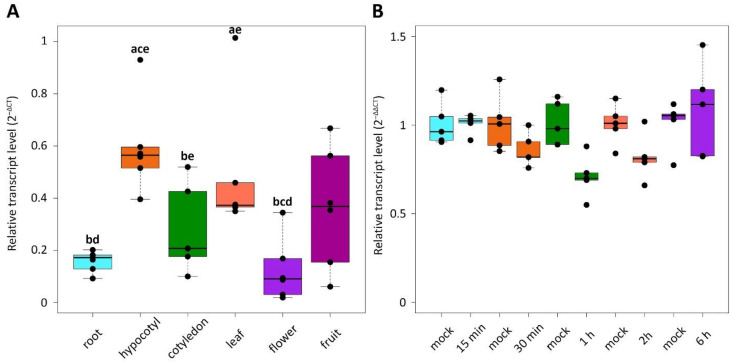
Relative transcript levels of *Cucumis sativus RALF34*. (**A**) Expression of *CsRALF34* in different plant organs. RT-qPCR analysis was performed using RNA isolated from the primary root tips of seven-day-old seedlings; hypocotyls and cotyledons of seven-day-old seedlings; the first true leaves of 14-day-old seedlings; flowers (male corollas and female corollas without ovaries) and fruits (ca. two weeks after pollination). Statistical analysis using an unpaired two-sample Wilcoxon test showed significant differences (*p* < 0.05) between organs: **a**—compared to the roots; **b**—compared to the hypocotyls; **c**—compared to the cotyledons; **d**—compared to the leaves; **e**—compared to the flowers. The *y* axis indicates the relative transcript level (2^−ΔCT^ method). (**B**) Expression of *CsRALF34* in response to treatment with 10 µM NAA. Four-day-old seedlings were incubated with NAA for 15 min, 30 min, 1 h, 2 h, and 6 h, respectively. Statistical analysis using the Wilcoxon test showed no significant differences (*p* > 0.05) between *CsRALF34* expression in mock-treated and NAA-treated roots. The *y* axis indicates the relative transcript level (2^−ΔΔCT^ method).

In order to study the expression levels of *CsRALF34* in different cucumber organs, we first analyzed transcriptomic data from the Cucurbit Expression Atlas in the Cucurbit Genomics Database v1. We found three studies evaluating cucumber gene expression either only in the roots (BioProject ID: PRJNA271595) [36] or in the roots and other organs (BioProject IDs: PRJNA80169, PRJNA312872) [37,38]. According to these data, *CsRALF34* was expressed not only in the roots but also in stems; in old and young leaves, including petioles; in tendrils; in male and female flowers; in unfertilized and fertilized ovaries; and in fruits (Figure S2). To validate these transcriptomic data, we performed the expression analysis of *CsRALF34* in different cucumber organs using RT-qPCR. *CsRALF34* was expressed in roots, hypocotyls, cotyledons, leaves, flowers and fruits (Figure 3A).

**Figure 4:**
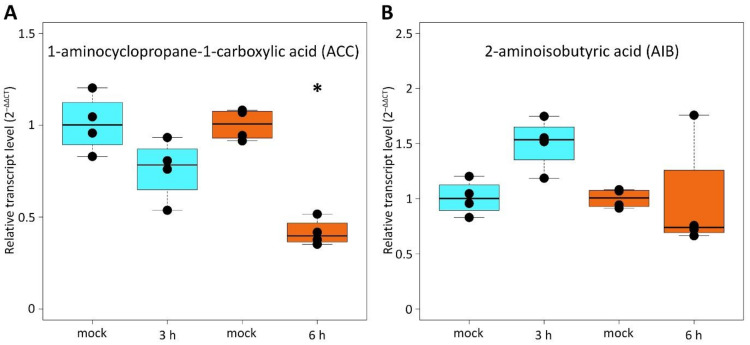
Expression of *Cucumis sativus RALF34* in response to 1-aminocyclopropane-1-carboxylic acid (ACC) (**A**) or 2-aminoisobutyric acid AIB (**B**) treatment. Four-day-old *C. sativus* seedlings were incubated either with 10 μM ACC for 3 h and 6 h, respectively, or with 1 μM AIB. RT-qPCR analysis was performed using RNA isolated from the apical centimeter of the primary roots. The *y* axis indicates the relative transcript level (2^−ΔΔCT^ method). Statistical analysis using the Wilcoxon test showed no significant differences (*p* > 0.05) between all samples except for roots treated with 10 μM ACC for 6 h (asterisk), where *CsRALF34* expression was reduced significantly (*p* < 0.05).

Experiments with the exogenous application of the synthetic auxin 1–naphthaleneaceticacid (NAA) at a concentration of 10 µM [8] for 15 min, 30 min, 1 h, 2 h, and 6 h to roots showed that *CsRALF34* expression in the roots was not affected by NAA (Figure 3B). However, the exogenous application of 10 μM 1-aminocyclopropane-1-carboxylic acid (ACC) significantly reduced *CsRALF34* expression levels in cucumber roots within 6 h, though not within 3 h (Figure 4A). On the other hand, the exogenous application of 1 µM of the ethylene biosynthesis inhibitor 2-aminoisobutyric acid (AIB) for 3 or 6 h had no effect on the expression levels of *CsRALF34* in roots (Figure 4B).

### 2.3. The First Cellular Events in Lateral Root Initiation in Cucumis sativus Roots

**Figure 5:**
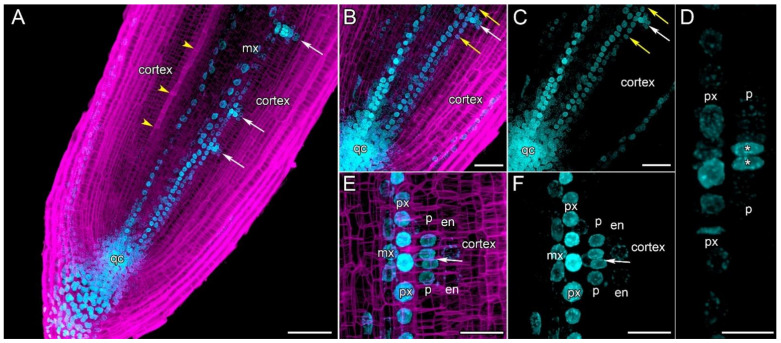
Auxin response maxima and lateral root primordia initiation in *Cucumis sativus* root meristem (*DR5::mRuby-H2B*). Confocal laser scanning microscopy of longitudinal vibratome sections. (**A**) Overview and (**B**,**C**) close-up of the parental root meristem show the auxin response maxima and initiation of the lateral root primordia (white arrows). Yellow arrowheads indicate the protophloem cell file. (**B**,**C**) Completed anticlinal division in the pericycle can be seen at a distance of 275 μm from the initial cells (white arrows). Yellow arrows point at the protoxylem. (**D**) The first anticlinal division was completed resulting in two pericycle founder cells with auxin response maxima at a distance of 275 μm from the initial cells (asterisks). (**E**,**F**) The second anticlinal divisions were completed in the pericycle founder cells (white arrow) and the first one in the endodermis. The primordium was initiated opposite a xylem pole. Note the auxin response maximum in the protoxylem. Maximum intensity projection of z-series: (**A**–**C**) of 25 optical sections, 24 µm in depth; (**D**–**F**) of 32 optical sections, 16 µm in depth. Blue channel, auxin response maxima (fluorescence of mRuby-H2B); magenta channel, SR2200-stained cell walls. En, endodermis; mx, metaxylem; p, pericycle; px, protoxylem; qc, quiescent centre. Scale bars: 100 µm in (**A**); 50 μm in (**B**,**C**); 20 μm in (**D**–**F**).

**Figure 6:**
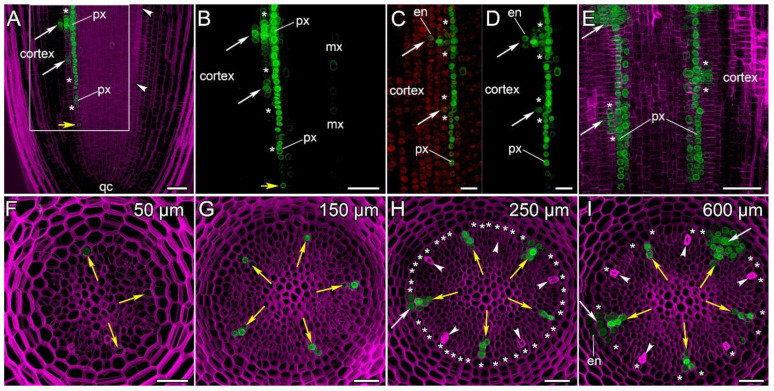
Localization of *RALF34* expression in *Cucumis sativus* root tips (*pCsRALF34::mNeonGreen-H2B)*. Confocal laser scanning microscopy of longitudinal (**A**–**E**) or cross (**F**–**I**) vibratome sections. (**A**) An overview and (**B**) close-up of the parental root meristem shows the acropetal sequence of *RALF34* promoter activity in the protoxylem and pericycle. *RALF34* expression arises first in the protoxylem at a distance of 126 µm from the initial cells (yellow arrows). No *RALF34* promoter activity was detected at the phloem poles (white arrowheads in A). (**B**–**E**) Establishment of *RALF34* expression in the protoxylem and metaxylem, pericycle layers, and the endodermis along the root meristem. The initiation of lateral root primordia is visible in the pericycle (white arrows). Two endodermal cells with local *RALF34* activity can be seen in the upper developing primordium. Arrows indicate the position of young lateral root primordia after the first anticlinal and periclinal divisions in the pericycle. (**E**) Lateral root primordia within the parental root meristem at a distance of 500–600 µm from the quiescent centre (qc). *RALF34* expression was localized in the protoxylem between primordia. (**F**–**I**) Establishment of *RALF34* expression in the protoxylem (yellow arrows), pericycle layers (asterisks), and the endodermis in the developing primordia (white arrows) on cross sections at a distance of 50–600 µm from the initial cells. No *RALF34* promoter activity is visible at the phloem poles (white arrowheads). Maximum intensity projection of z-series: (**A**,**B**) of 46 optical sections, 40 µm in depth; (**C**,**D**) of 12 optical sections, 26 µm in depth; (**E**) of 40 optical sections, 15 µm in depth; (**F**–**I**) of 50 optical sections, 42 µm in depth. Green channel, the fluorescence of mNeonGreen-H2B; magenta channel, SR2200-stained cell walls; red channel (**C**,**D**), nuclei stained with DAPI. Asterisks indicate developing pericycle; en, endodermis; mx, metaxylem; px, protoxylem; qc, quiescent centre. Scale bars: 50 µm in (**A**,**B**,**E**), 20 μm in (**C**,**D**), and 30 μm in (**F**–**I**).

The first events during lateral root initiation in cucumber were studied on longitudinal sections of transgenic hairy roots harboring a *DR5::mRuby-H2B* construct for the visualization of auxin response maxima (Figure 5). The primordia were initiated opposite the xylem poles at a distance of 200–300 µm proximal to the initial cells in the parental root meristem (Figure 5A–C). It is important to note that protophloem differentiation takes place at this distance, and accordingly, the first sieve elements were visible (Figure 5A). The earliest lateral root initiation event detectable at the histological level was symmetric formative anticlinal division in one pericycle cell opposite the protoxylem, resulting in the formation of two cells with auxin response maxima (Figure 5D). The cells of the proto- and metaxylem files at this distance from the initials maintain an auxin response; however, this response is maximal in the two protoxylem cells adjacent to the two pericycle cells, resulting from the first formative division (Figure 5D–F; Video S1). The protoxylem cells at this distance maintain the ability to proliferate, too (Figure 5D). Importantly, the formation of founder cells occurred within the three files of the pericycle cell layer (Figure 6H). Endodermal layers became involved in primordia formation after the completion of the second anticlinal divisions of two pericycle founder cells at a distance of approx. 350–400 µm from the initials (Figure 5E,F; Video S1). Auxin response maxima in the endodermis were established, like in the pericycle, always simultaneously in two cells.

### 2.4. Localization of RALF34 Expression in the Meristem of Cucumis sativus Roots

The expression pattern of *CsRALF34* was studied on longitudinal and cross sections of transgenic cucumber hairy roots harboring a *pCsRALF34::mNeonGreen-H2B* construct (Figure 6). The promoter activity of *CsRALF34* in the parental root meristem was visualized first in the protoxylem cell file at a distance of approximately 90–150 µm from the initial cells (Figure 6A–D,F,G) before any cellular differentiation and persisted throughout the protoxylem files to the end of the root meristem (Figure 6E,I). Then, *CsRALF34* promoter activity could be observed in cell files of the peripheral metaxylem (Figure 6B,G,H). Additionally, only then, at a distance of approximately 200–250 µm from the initial cells, *CsRALF34* expression commenced in certain single pericycle cells at the xylem pole (Figure 6B–D,H). Promoter activity was maintained along the pericycle only in the primordia themselves and in the cells adjacent to the primordia (Figure 6B–E). The establishment of *CsRALF34* expression in the endodermis was connected with primordium development and the involvement of the endodermis in this process (Figure 6C–E,I). In the endodermis, *CsRALF34* expression was induced only in the two cells adjacent to the dividing cells of the pericycle (Figure 6C,D). When the inner layers of the cortex became involved in the formation of the lateral root primordium, *CsRALF34* expression was induced in them (Figure 6E). *CsRALF34* promoter activity was maintained in the lateral root primordia until the beginning of the elongation zone in the parental root. No *CsRALF34* promoter activity was visible in the pericycle or endodermis at the phloem poles (Figure 6A,H,I)*. CsRALF34* expression always commenced in the pericycle prior to the formation of the auxin response maxima in the two founder cells (Figure 5A–C and Figure 6A–D).

### 2.5. CsRALF34 Peptide Accumulated in the Apoplast and Along the Cell Walls of Root Cortex Cells

**Figure 7:**
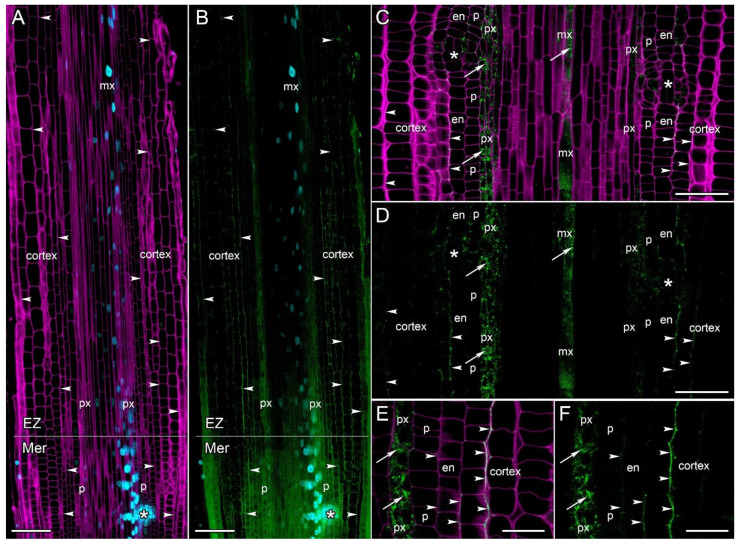
Localization of the RALF34 fusion protein in *Cucumis sativus* root tips (*pCsRALF34::CsRALF34-mNeonGreen, DR5::mRuby-H2B*). Confocal laser scanning microscopy of longitudinal vibratome sections. (**A**,**B**) The fusion of the fluorescent tag *mNeonGreen* with the *Cs*RALF34 peptide co-localized with auxin response maxima (as signified by *DR5* activity). (**A**) Overview and (**C**–**F**) close-ups of the basal part of the meristem (Mer) and the elongation zone (EZ) show the translation of CsRALF34 in the xylem and lateral root primordia and the accumulation of fusion protein in the apoplast and along the cell walls of root cortex cells (white arrowheads). (**C**,**D**) Translation of *CsRALF34* in xylem files (white arrows) and lateral root primordia, transport and accumulation of fusion protein in the apoplast and cell walls of the cortex (white arrowheads). (**E**,**F**) A close-up of the protoxylem–cortex in the apical part of the elongation zone. Localization of *Cs*RALF34 in the protoxylem (white arrows), transport and accumulation of the fusion protein to the apoplast and cell walls of cortex cells (white arrowheads). Green channel, the fluorescence of mNeonGreen; magenta channel, SR2200-stained cell walls; blue channel (**A**,**B**), auxin response maxima (*DR5*). Single optical sections. Asterisks (*) indicate developing lateral root primordia; en, endodermis; mx, metaxylem; p, pericycle; px, protoxylem. Scale bars: 100 µm in (**A**,**B**); 50 μm in (**C**,**D**); 20 μm in (**E**,**F**).

**Figure 8:**
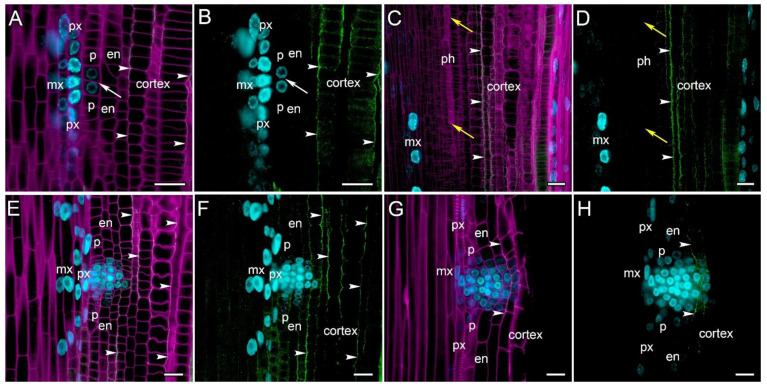
Initiation of lateral root primordia and localization of *Cs*RALF34 fusion protein in *Cucumis sativus* root meristem (*pCsRALF34::CsRALF34-mNeonGreen, DR5::mRuby-H2B*). *Cs*RALF34 fusion protein was detected in the apoplast and along the cell walls of root cortical cells (white arrowheads). Confocal laser scanning microscopy of longitudinal vibratome sections. (**A**,**B**) Initiation of lateral root primordia at a distance of 260 µm from the initial cells. Two founder cells are present in the pericycle (arrow). *Cs*RALF34 fusion protein accumulation can be seen in the apoplast along the cortical cells only. Note the auxin response maximum in the protoxylem. (**C**,**D**) The phloem pole in the parental root meristem at a distance of 200–300 µm from the initial cells. No *CsRALF34* expression can be detected in the protophloem cell file and phloem parenchyma (yellow arrows). (**E**,**F**) Developing lateral root primordium at a distance of 700 µm from the initial cells. *Cs*RALF34 fusion protein accumulation can be detected in the apoplast along the cortical cells. (**G**,**H**) Developing lateral root primordium at a distance of 1200 µm from the initial cells. *Cs*RALF34 fusion protein has mostly disappeared from the apoplast of the cortical cells. (**A**,**B**,**E**–**H**) Single optical sections. (**C**,**D**) Maximum intensity projection of z-series: of 25 optical sections, 20 µm in depth. Green channel, the fluorescence of mNeonGreen; magenta channel, SR2200-stained cell walls; blue channel, auxin response maxima (*DR5*). En, endodermis; mx, metaxylem; p, pericycle; px, protoxylem. Scale bars: 20 µm.

The tissue distribution and cellular localization of the CsRALF34 peptide were studied on the longitudinal of transgenic cucumber roots harboring a *pCsRALF34::CsRALF34-mNeonGreen* construct with or without the *DR5::mRuby-H2B* construct for the visualization of auxin response maxima (Figure 7 and Figure 8). The *CsRALF34* CDS was fused to that of *mNeonGreen* either via a linker or without a linker (Figure S3). The fusion construct without the linker showed the ability of the fusion protein to act (be transported) in a non-cell-autonomous manner (Figure 7, Figure 8 and Figure S3A–D). Roots carrying the construct for auxin response maxima visualization (Figure 7A,B) or not (Figure 7C–F) showed the same pattern of the distribution of the *Cs*RALF34 fusion protein.

The translation of the *Cs*RALF34 fusion protein was visualized in the protoxylem, metaxylem, and pericycle part of primordia from the meristem to the elongation zone of cucumber roots (Figure 7). The translation pattern of the *Cs*RALF34 fusion protein completely coincided with the visualization of its promoter activity (Figure 6 and Figure 7). The *Cs*RALF34 fusion protein was transported to and accumulated in the apoplast and along the cell walls of root cortex cells in the basal part of the meristem and in the elongation zone (Figure 7). Synthesis and subsequent accumulation of the *Cs*RALF34 fusion protein were not related to the auxin response maxima in roots (Figure 7A,B and Figure 8A–D).

We also especially analyzed the *Cs*RALF34 protein pattern in the context of the lateral root primordia initiation and development (Figure 8). The initial stages of lateral root initiation were identified by the first anticlinal divisions in the pericycle, accompanied by the auxin response maxima formation at a distance of 200–300 µm from the initial cells (Figure 8A,B). *Cs*RALF34 protein was accumulated in the apoplast and along the cell walls of the middle cortex files. No *Cs*RALF34 protein accumulation was visualized in the apoplast of the pericycle and endodermis in the primordium initiation zone (Figure 8A,B). No peptide synthesis was visualized at the phloem pole, but its accumulation also occurred in the apoplast and along the cell walls of the cortex files (Figure 8C,D). During the primordia development in the basal part of the meristem, peptide synthesis is visualized in the protoxylem and pericycle, and its transport and subsequent accumulation occur in the cell walls of the cortex files (Figure 8E,F). In the basal part of the elongation zone, the *Cs*RALF34fusion protein has mostly disappeared from the apoplast of the cortical cells (Figure 7A,B and Figure 8G,H). Interestingly, in this zone, in the apoplast of primordial cells with a cortical origin, an accumulation of the *Cs*RALF34 protein was retained (Figure 8G,H). Above the elongation zone, in the differentiation zone, neither the *Cs*RALF34 peptide synthesis nor the apoplast accumulation was not visualized.

### 2.6. The Expression of CsTHE1 Takes Place in All Cells of Cucumis sativus Root Tips

**Figure 9:**
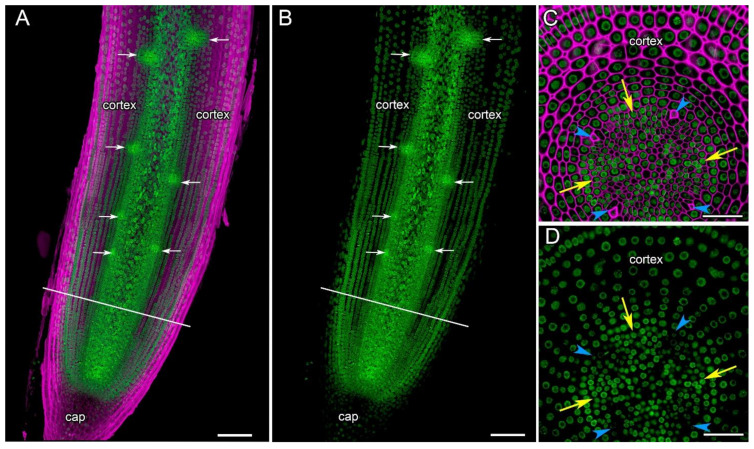
Localization of *THESEUS1* expression in *Cucumis sativus* root tips (*pCsTHESEUS1::mNeonGreen-H2B*). Confocal laser scanning microscopy. (**A**,**B**) Longitudinal section of the parental root meristem with developing lateral root primordia (white arrows). (**C**,**D**) Cross section at the distance of 260 μm from the initial cells (see white lines in (**A**,**B**)). Phloem poles (protophloem cells) are indicated by blue arrowheads, and xylem poles by yellow arrows. (**A**,**B**) Maximum intensity projection of z-series of 10 optical sections, 12 µm in depth. (**C**,**D**) Single optical sections. Green channel, the fluorescence of mNeonGreen; magenta channel, SR2200-stained cell walls. Scale bars: 100 µm in (**A**,**B**); 50 μm in (**C**,**D**).

The analysis of the longitudinal and transverse sections of transgenic cucumber roots containing the *pCsTHE1::mNeonGreen-H2B* insert has shown that *CsTHE1* expression was localized in the initial cells, the root cap cells, central cylinder, primary cortex, and the rhizodermis (Figure 9). Throughout the root tip, the expression of the *CsTHE1* gene was maintained in cell files of the central cylinder, including the xylem and phloem poles, and in all primary cortex cells (Figure 9C,D), including the cells between the developing lateral root primordia (Figure 9A,B). In addition, the expression of *CsTHE1* was observed in lateral root primordia from their earliest stages of development onwards and was maintained in all primordia cells.

Thus, the localization of the *CsTHE1* promoter activity in cucumber showed that the expression of *CsTHE1* occurs in all cells of cucumber root tissues in all transgenic root segments. The intensity of the fluorescence of the reporter suggests the similarity of the expression levels of this gene in all root tip tissues (Figure 9).

## 3. Discussion

The aim of this study was the comparison the role of the small signaling peptide *Cs*RALF34 and its receptor *Cs*THESEUS1 in the lateral root formation of cucumber with the role of *At*RALF34 (RALF-LIKE34, *At*RALFL34) in lateral root formation in *Arabidopsis*. This required us to first ensure that *Cs*RALF34 is indeed the ortholog of *At*RALF34. The number of the Arabidopsis RALF peptides is still controversial, as it varies between 34 and 39 [12]. We used the sequences of 37 Arabidopsis RALF peptides for searches in the cucumber proteome because it is difficult to determine whether the last two Arabidopsis RALF peptides, *At*RALF36 (ID: AT2G32785) and *At*RALF37 (ID: RALF37), are in fact members of the RALF family [12]. The phylogenetic analysis of all 37 Arabidopsis RALF peptides [12], together with all identified *C. sativus* RALF peptides, resulted in the identification of 17 members of the cucumber RALF family. Of these, 13 members had been identified in cucumber previously [5], and we identified four new cucumber RALF peptides. Among the 37 *Arabidopsis* RALF peptides, there are two whose function as RALF peptides is somewhat controversial, namely AT2G32890 and AT4G14020, previously annotated as *At*RALF17 and *At*RALF35, respectively [12]. Two of the four newly identified cucumber RALFs, Csa5G054060 and Csa6G511800, map closely to *At*RALF32 and to the controversial AT4G14020. Furthermore, the bootstrap value for this subclade was lower than 50%. Therefore, we propose that the number of peptides in the cucumber RALF family should be revised from 17 to 15. The phylogenetic tree makes it difficult in several cases to assign cucumber RALF peptides to their putative Arabidopsis orthologs (see, e.g., AtMEDOS1–AtMEDOS4); nevertheless, a single cucumber *At*RALF34 ortholog, named *Cs*RALF34, was identified (Figure 1).

All mature RALF34 peptides of Cucurbitales display very high identity with the *At*RALF34 mature peptide, suggesting that, like *AtRALF34,* Cucurbitales RALF34 is perceived by the receptor kinase THESEUS1 [21]. *At*THE1 belongs to the *Catharanthus roseus* RLK1-like receptor-like kinases (*Cr*RLK1-like RLKs) family comprising the 17 members [39]. Cucumber *Cr*RLK1-like RLKs are represented by 21 proteins, i.e., the family contains four more proteins than its counterpart in Arabidopsis [39]. A single *At*THESEUS1 ortholog was identified in cucumber and named *Cs*THESEUS1 (*Cs*THE1) (Figure 2).

The next step was to compare the regulation of the expression of *CsRALF34* with that of *AtRALF34*. Data on the broad range of *CsRALF34* cucumber organ-specific expression described in cucumber transcriptomics studies [37,38] were confirmed by our RT-qPCR data: *CsRALF34* is expressed in all plant organs examined.

It had also been reported that *AtRALF34* belongs to the late auxin-responsive genes in that the expression of *AtRALF34* was downregulated significantly after six hours of treatment of *Arabidopsis* seedlings with either 5 or 10 μM NAA and significantly upregulated after 24 h of treatment with the same NAA concentrations [8]. Nevertheless, Omelyanchuk et al. [40] showed that *AtRALF34* does not belong to the late auxin-responsive genes in that its expression levels do not change after the treatment of Arabidopsis seedlings with 1 μM IAA for 6 h. Moreover, the meta-analysis of the 22 available Arabidopsis gene expression datasets showed that *AtRALF34* does not fall into the group of early auxin-responsive genes [41]. Our RT-qPCR data confirmed that *CsRALF34* does not belong to either early or late auxin-responsive genes; the expression of *CsRALF34* did not change after the treatment of cucumber roots with 10 μM NAA for 15 min, 30 min, 1 h, 2 h or 6 h (Figure 3). We did not test longer treatments in order to avoid measuring secondary effects. Altogether, *CsRALF34* seems to differ from *AtRALF34* in that its expression is not regulated by auxin.

A luciferase (LUC) assay conducted in protoplasts containing a *pAtRALF34::LUC* construct showed that LUC activity was significantly downregulated in the presence of either Ethylene-Responsive Transcription Factor 4 (ERF4) or ERF9 [8]. Therefore, we analyzed the regulation of *CsRALF34* expression via the ethylene signaling pathway. The expression of *CsRALF34* was not changed by treatment with 1 μM AIB, a competitive inhibitor of the conversion of 1-aminocyclopropane-1-carboxylic acid to ethylene [42], for 3 h or 6 h, whereas it was significantly downregulated by treatment with 10 μM ACC, the direct precursor of ethylene [43], for 6 h (Figure 4). In summary, the data of Murphy et al. [8] and our data may indicate only the partial involvement of ethylene in the regulation of the expression of *AtRALF34*, as well as *CsRALF34*.

Previously, we have shown in detail the sequence of events involved in the initiation and development of lateral root primordia in squash (*Cucurbita pepo*, Cucurbitaceae) (Ilina et al., 2018). A similar pattern of lateral root initiation was observed in cucumber roots (Figure 5 and Figure 8A,B). A significant difference between both plants is the presence of only one pericycle layer in cucumber. A local auxin response maximum is created in the four protoxylem cells at a distance of about 200 µm from the initial cells (Figure 5). This determines the location of the new primordium both along the longitudinal axis and in the radial position (Figure 5D–F and Figure 6H,I). In Arabidopsis, founder cells in the pericycle are recruited by a multi-step auxin-dependent process opposite this local auxin maximum in the protoxylem [44]. A similar recruitment process occurs in cucumber roots in the endodermal cells and several inner cortical layers (Figure 5 and Figure 8).

Despite our extensive knowledge of the lateral root initiation processes in Arabidopsis, in the case of cucurbits, many questions remain unexplained regarding the mechanisms of primordia initiation. For example, lateral root priming in cucumber and squash is difficult to explain on the basis of both the ‘root clock’ theory [45,46,47] and the ‘reflux–and–growth’ theory [48]. For terminology, see the review by Laskowski and ten Tusscher [49]. First of all, we would like to draw attention to the lack of a temporal and/or spatial gap between the formation of a local auxin maximum in the cucumber protoxylem cells (prebranch site formation), the priming of the pericycle cells in the meristem (founder cell identity establishment), and the initiation of lateral root primordia from the founder cells, as has been shown for Arabidopsis [46,49]. Our data show that auxin-dependent tissue recruitment during primordia formation always occurs in a centrifugal direction (Figure 5 and Figure 8). The formation of the auxin response maximum occurs sequentially from the protoxylem to the cortical cells rather than vice versa. Like De Smet et al. (2007) [46], we also used DR5 or DR5v2 [50] as a reporters of cellular response to auxin; here, like in Arabidopsis, it also demonstrated the presence of auxin maxima in the root caps of lateral roots. Destruction of the lateral root cap cells with the possible release of auxin [51,52] occurs in cucumber roots much higher as the initiation zone of lateral root primordia in the meristem of the parental root (Figure 7A,B). Unfortunately, the *DR5::Luciferase* assay [51,53] is difficult to apply to cucumber roots because all lateral root initiation processes are located in an area no more than 250 µm from the quiescent centre.

The number of founder cells in the pericycle, total or in each cell file, from which the lateral root primordium is formed is hotly debated [29,31,44,54,55]. In the cucumber root, only one cell in each pericycle cell file will become a founder cell. After anticlinal division, two cells with auxin response maxima are formed (Figure 5D and Figure 8A,B), as we have shown earlier for squash [31]. The auxin response maximum is formed in this single founder cell directly during its anticlinal division, as has been shown by in vivo observations on Arabidopsis roots [34]. The two daughter cells always undergo at least one more anticlinal division (Figure 5E,F) before proceeding to periclinal divisions and primordium formation (Figure 8E,F). Similar events take place in the endoderm and several inner cortical layers as they undergo auxin-dependent recruitment (Figure 8G,H). Apparently, cucurbits lack nuclear migration and asymmetrical cell divisions (Figure 5D and Figure 8A,B), typical for lateral root initiation above the elongation zone in plants like Arabidopsis [34].

Recently, Torres-Martínez et al. [29] has suggested the presence of two types of lateral root initiation in Arabidopsis: unicellular longitudinal (from one founder cell) and bicellular longitudinal (from two neighboring founder cells). This is common to all plant species in which the lateral root initiation occurs in cells that have completed the elongation [30,55,56,57,58]. However, as far as we can say based on squash and cucumber, when a lateral root is initiated directly in the meristem of the parental root, the pericycle cells are short, and the lateral root initiation always takes place from only single founder cell in the pericycle cell file.

The pattern of *AtRALF34* expression in Arabidopsis roots using a *pRALFL34*::*n3xRFP* construct with a promoter length of 869 bp was previously described [8,13]. *AtRALF34* was expressed in the pericycle on the xylem pole behind the elongation zone before any visible sign of asymmetric anticlinal cell divisions; in the pericycle of the lateral root initiation site, but also in the flanking cells; and in the epidermis [8]. *AtRALF34* expression was also detected in the entire root cap and in the epidermis of the root meristem, and of the elongation zone [13]. However, the tissue distribution pattern of the *At*RALF34 peptide was not presented. This is relevant as RALFs, like other signal peptides, such as CEPs, are known to act non-cell-autonomously [17].

Our reporter assays data have shown that the *RALF34* expression pattern in cucumber roots is somewhat different from that in Arabidopsis (Figure 6). First of all, *CsRALF34* expression begins in the protoxylem cell file alone and only then commences in the founder cells and the lateral root primordia, including the flanking cells, the endodermal and cortical derivatives (Figure 6B–E,I). *CsRALF34* expression in the single founder cell in the pericycle appears to start before the local auxin maximum is formed (Figure 6C,D). *CsRALF34* expression was not detected in the root cap and the epidermis across the entire root, nor in all tissues behind the elongation zone (Figure 6A). Interestingly, *CsRALF34* expression occurs only at the xylem pole (Figure 6A,F–I) and begins in the protoxylem at a distance of about 120 μm from the initials before protophloem differentiation (Figure 6A,F–H).

In this study, we have shown for the first time the tissue distribution and accumulation of the *CsRALF34* gene product along the cucumber root tip (Figure 7 and Figure 8). *Cs*RALF34 fusion protein was possibly detected in the endoplasmic reticulum and the Golgi complex (based on RALF3 subcellular localization in *Solanum chacoense* [17]) of the protoxylem, metaxylem and primordia cells (Figure 7C–F), which agrees well with the data on the expression of this gene in roots (Figure 6). Then *Cs*RALF34 fusion protein accumulated in the apoplast along the cell walls of root cortex cells on the xylem or phloem pole (Figure 7 and Figure 8). No *Cs*RALF34 protein accumulation was observed in the pericycle during the early stages of primordium development (Figure 8A,B) or behind the elongation zone (Figure 7A,B). However, mRNA transport into the shoot cannot be excluded [14]; but we have not detected *Cs*RALF34 mature fusion protein in the root differentiation zone and above.

The expression of *Cs*RALF34 in the pericycle is clearly a marker of the early events of lateral root initiation, i.e., founder cell specification. The initiation of its expression in the pericycle cell appears to occur earlier than the expression of *C. pepo GATA24*, the *AtGATA23* orthologue [32]. This is consistent with the results of Murphy et al. [8], which implies that *AtRALF34* acts genetically upstream of *AtGATA23*. However, our data show that the product of *CsRALF34* does not accumulate in the apoplast of the pericycle and young primordium cells and is most likely not involved in the regulation of lateral root initiation in cucumber.

*CsTHE1* was expressed constitutively in all cucumber root tissues, including the root cap (Figure 9). This pattern differs from the distribution of *AtTHE1* expression in *Arabidopsis* roots, where based on a GUS reporter fusion, it was present in the elongating cells and vascular tissues but not in the meristem or cortex cells of the differentiation zone [23]. There is every reason to believe that the *Cs*RALF34 receptor itself consists of several domains, and *Cs*THE1 is its putative constitutive part which can dimerize with other members of this receptor family, for example, with FERONIA or other co-receptors, such as LLG [59]. Ectopic lignification in the basal part of the meristem and the beginning of the elongation zone may also be mediated by RALF34–THE1 [22].

## 4. Materials and Methods

### 4.1. Plant Material and Bacterial Strains

Cucumber (*Cucumis sativus* L.) cv. Kustovoy (Sortsemovosch, Saint Petersburg, Russia) was used in this study. *Escherichia coli* strain XL-1 Blue was used for molecular cloning. *Rhizobium rhizogenes* (formerly *Agrobacterium rhizogenes*) strain R1000 was used for the genetic transformation of cucumber seedlings. The R1000 strain contains the pRiA4b Ri-plasmid derived from the *R. rhizogenes* A4 strain [60,61].

### 4.2. Phylogeny and Bioinformatics

All known *Arabidopsis thaliana* sequences of RALF proteins [12], as well as their receptors from the *Catharanthus roseus* receptor-like kinase 1-like (*Cr*RLK1L) family [39], were downloaded from the *Arabidopsis* Information Resource (TAIR, www.arabidopsis.org, accessed on 1 May 2023) [62] and used as queries to find the homologous amino acid sequences of *C. sativus* cv Chinese Long v.2 [37] in the Cucurbit Genomics Database v1 (CuGenDB v1, cucurbitgenomics.org, accessed on 1 May 2023) [63]. The RALF amino acid sequences from another cucumber cultivar, Gy14 v.1 [64], identified previously [5], were also downloaded from CuGenDB v1 and subsequently used for alignments. All alignments were performed using online Clustal Omega software (www.ebi.ac.uk/Tools/msa/clustalo/, accessed on 1 May 2023) [65] at default settings. The alignment file was transferred into the MEGA7.0 software [66], followed by phylogenetic tree construction using the Maximum Likelihood (ML) method [67].

The MEGA7.0 options for phylogenetic tree building were as follows. The Jones–Taylor–Thornton (JTT) model [68] with the evolutionary rate differences among sites (+G parameter) (Yang, 1994) and with the proportion of invariable sites (+I parameter) [69] was used for RALF proteins phylogeny reconstruction (Figure 1). The Whelan and Goldman +Freq. model [70] with the evolutionary rate differences among sites (+G parameter) was used for CrRLK1-like RLKs proteins phylogenetic analysis (Figure 2). These models were chosen from the list of models with the lowest Bayesian information criterion (BIC) scores [71]. Models with the lowest BIC scores are considered to best describe the substitution pattern. The number of discrete gamma categories was equal to 2. The “use all sites” option was chosen for gaps/missing data treatment. The tree inference options were used as follows. Nearest-Neighbor-Interchange was used as ML heuristic method. Neighbor-joining (NJ) [72] and BioNJ [73] algorithms were chosen for building the initial tree for ML. Phylogeny was tested using the bootstrap method with 1000 replicates for RALF and with 500 replicates for *Cr*RLK1-like RLKs proteins.

The sequence logo was created based on the Clustal Omega alignment of the RALF34 proteins from 20 Cucurbitales species using WebLogo (weblogo.berkeley.edu/, accessed on 1 May 2023) [74]. Information about Cucurbitales RALF34 amino acid sequences, together with all databases used and appropriate references, are listed in Table S1. In all cases, both Arabidopsis RALF34 amino acid and cucumber RALF34 amino acid or CDS sequences were used as queries. For the 16 members of the Cucurbitales with available annotated genomes, CDS and amino acid sequences were used in BLASTN, TBLASTN and BLASTP algorithms. For the other four members of the Cucurbitales for which no proteome sequences (only genome assemblies) were available, BLASTN and TBLASTN algorithms were used, followed by in silico translation of the identified RALF34 CDS using the online ExPASy translate tool (web.expasy.org/translate, accessed on 1 May 2023) [75].

### 4.3. Molecular Cloning, Plasmid Construction and Plant Transformation

A series of genetic constructs containing promoter–reporter fusion cassettes were developed using multisite Gateway technology (Gateway LR Clonase II Plus, Thermo Fisher Scientific, Waltham, MA, USA) (Table 1 and Table S2) [76].

Two different destination binary vectors were used: pKGW-RR-MGW, carrying the RedRoot (RR) screening cassette *pAtUBQ10::DsRED1* [77] in the backbone, and pKGW-DR-MGW (Figure S4), carrying the auxin-responsive cassette *DR5::mRuby3-H2B* (DR) in the backbone. To develop pKGW-DR-MGW, the first step was to avoid *Kpn*I sites in entry vectors. Therefore, the *DR5* promoter [78] was cloned into pDONR P4-P1R via the Gateway BP clonase reaction (Thermo Fisher Scientific, Waltham, MA, USA) and *mRuby3-H2B* was *Bam*HI*-Not*I cloned into pUC18-entry8 [79]. pENTRattR2attL3-TermAct [32] was used as a donor of the *A. thaliana Actin2* gene terminator (*TermAct*). The intermediate construct pKGW-RR-MGW-*DR5::mRuby3-H2B-TermAct* was assembled by multisite Gateway technology. The *DR5::mRuby3-H2B-TermAct* cassette was PCR-amplified and cloned into the *Kpn*I site of the pKGW-MGW backbone, resulting in pKGW-DR-MGW. The insert position relative to the attR3 site and prospective cassette assembled by Gateway cloning is “tail to tail”.

To create all required binary vectors, a series of entry vectors were generated. A list of plasmids and vectors used for entry vector construction is given in Table S3. Two promoter-containing entry vectors were developed. The upstream sequence containing the putative promoter region and 5′-UTR (2443 bp, from −2479 to −36 bp before the predicted translational start site) of the *C. sativus RALF34* gene identified in this study was PCR-amplified using the genomic DNA of cucumber as a template and cloned into pENTRattL4attR1_BSAI (Wageningen University, Wageningen, The Netherlands) using the *Sma*I restriction site. A 6000 bp fragment containing promoter and coding region of the *C. sativus THESEUS1* gene was PCR-amplified using the genomic DNA of cucumber as a template, cloned into the pJET1.2 vector (Thermo Fisher Scientific), and its sequence was verified. The upstream sequence containing the putative promoter region and 5′-UTR (2930 bp, from −3181 to −251 bp before the predicted translational start site) of the *CsTHESEUS1* gene was PCR-amplified using pJET1.2-*CsTHESEUS1* as a template and cloned *Xho*I*-Kpn*I into pENTRattL4attR1_BSAI.

Overlap extension PCR was used to generate fusions of *CsRALF34* and *mNeonGreen* coding sequences via a linker sequence or without it. *CsRALF34* was PCR-amplified using cucumber genomic DNA as a template, cloned into a pJET1.2 vector, and its sequence was verified. *CsRALF34*-pJET1.2 vector and a commercial plasmid harboring the *mNeonGreen* sequence (Allele Biotechnology plasmid #H2B-213 [80]) were used as templates in overlap extension PCR. The resulting *CsRALF34-linker-mNeonGreen* and *CsRALF34-mNeonGreen* fusions were cloned *Kpn*I-*Not*I into the pUC18-entry8 vector.

The destination binary vectors pKGW-RR-MGW and pKGW-MGW were kindly provided by Erik Limpens (Wageningen University, Wageningen, The Netherlands). LR plus clonase reactions were prepared according to the manufacturer’s instructions, using the entry and destination vector combinations listed in Table S2. The *mNeonGreen-H2B*-pUC18-entry8 entry vector had been described previously (Kiryushkin et al., 2019) [32]. The pENTRattR2attL3-*TermAct* or pENTRattR2attL3-*T35S* (Wageningen University, The Netherlands) constructs were used as donors of the *TermAct* or *T35S* terminators in all reactions.

All resulting fusions in all constructs were verified by the PCR amplification of fragments and sequencing of the products. All primer sequences are given in Table S4, and the combinations of primers for different cloning steps are given in Table S5. All binary vectors were transferred into agrobacterial cells by an electroporation [81]. *Rhizobium (Agrobacterium) rhizogenes*-mediated plant transformation was carried out as described previously [32,81] with minor modifications. Transformants (composite plants) were cultured in polypropylene vessels (OS140BOX, Duchefa Biochemie, Haarlem, The Netherlands) filled with autoclaved vermiculite moistened with 4× Hoagland’s medium [82] or in an aeroponic system with ½ strength Hoagland’s medium at 25 °C and a 16 h light period in an MLR-352H climate chamber (Panasonic, Osaka, Japan). Water mist in the aeroponic system was generated by a Defensor 505 air humidifier (Condair, Garching-Hochbrück, Germany). With each construct, at least two independent transformation series (biological replicates) were performed on 30 plants each. 10–15 Transgenic roots 8–10 cm in length were harvested 5–7 times with 6–7-day intervals for each variant.

### 4.4. Plant Organs Collection

Six types of cucumber organs were collected: the primary root representing 10 mm apical segments of seven-day-old seedlings; hypocotyls and cotyledons of seven-day-old seedlings; the first true leaves of 14-day-old seedlings; flowers (male corollas together with female corollas without ovaries) and fruits (about two weeks after pollination). Samples were flash-frozen in liquid nitrogen. Frozen plant material was stored at −80 °C.

### 4.5. Treatments with Exogenous Auxin, Precursor of Ethylene and Inhibitor of Ethylene Biosynthesis

Four-day-old wild-type cucumber seedlings with 5–7 cm long roots were incubated either with 10 μM 1-naphthaleneacetic acid (NAA) (N0640, Sigma-Aldrich, Saint Louis, MO, USA) or with 10 μM of the ethylene precursor 1-aminocyclopropane-1-carboxylic acid (ACC) (A3903, Sigma-Aldrich), or with 1 μM of the inhibitor of ethylene biosynthesis, 2-aminoisobutyric acid (AIB) (850993, Sigma-Aldrich). Seedlings were incubated in aerated 1× Hoagland’s medium, supplemented either with NAA for 15 min, 30 min, 1 h, 2 h and 6 h; or ACC for 3 h and 6 h; or AIB for the same time. The concentrations and periods of exposition used in this study were selected based on data reported previously for NAA [8,31,32,83], ACC or AIB [84,85,86]. Each experiment included at least 35–42 seedlings, and five independent replicates were used for each time point. The first apical segments, 10 mm long, were collected and flash-frozen in liquid nitrogen. Frozen plant material was stored at −80 °C.

### 4.6. RT-qPCR Assays

Total RNA was extracted from frozen plant material using the ExtractRNA reagent (Evrogen, Moscow, Russia). RNA quantity and integrity were measured using a Qubit 4.0 fluorimeter (Thermo Fisher Scientific) using the Qubit RNA BR Assay and IQ Assay Kits subsequently. Reverse transcription was performed using the Maxima First Strand cDNA synthesis kit for RT-qPCR with dsDNase (Thermo Fisher Scientific), as described previously [32]. One μL of cDNA from a non-diluted sample (total volume 20 μL) was used for each qPCR reaction.

The RT-qPCR analysis was performed using the Quant Studio 5 Real-Time PCR system (Thermo Fisher Scientific). Each qPCR reaction was carried out in a total volume of 20 μL. Detection based on SYBR Green I dye chemistry was used: both for auxin-treated root samples (Maxima SYBR Green/ROX qPCR master mix (2×), Thermo Fisher Scientific) and for different organ samples or ACC/AIB treated root samples (qPCRmix-HS SYBR+LowROX, Evrogen). PCR conditions were the same as described previously for cucumber [32] except for the melting curve stage: the reheating of PCR products was performed at 95 °C, followed by a decrease in the temperature to 60 °C. Then, the temperature was increased gradually from 60 to 95 °C in steps of 0.15 °C per s. Primers used for qPCR are listed in Table S6. Primers were designed using the Vector NTI Advance v 11.0 software (Thermo Fisher Scientific). Purified PCR primers were purchased from Evrogen (Moscow, Russia).

Quantification cycles (Cq) were determined using the Quant Studio Design and Analysis software v. 1.5.1 (Thermo Fisher Scientific). Relative transcript levels were calculated using the 2^−ΔΔCT^ and 2^−ΔCT^ methods for experiments with or without a control group, respectively [87,88]. PCR efficiency for all primer pairs was between 93 and 98%. Elongation factor *EF1α* was chosen as a reference gene for RT-qPCR data normalization based on literature data concerning the stability of reference gene expression in cucumber roots [89].

### 4.7. Fluorescence Protein Reporter Assays and Microscopy

The screening of transgenic roots was performed based on the fluorescence either of DsRED1 or mRuby3 reporters (Table 1) under a SteREO Lumar.V12 fluorescent stereomicroscope (Carl Zeiss, Oberkochen, Germany) [76,81,90,91]. The filter set 43 HE (EX BP 550/25, EM BP 605/70, Carl Zeiss) for the observation of DsRED1 or mRuby3 fluorescence was used.

For the localization of mNeonGreen and mRuby3 reporters, 7–9 mm long tips or the segments of transgenic hairy roots of cucumber were fixed and sectioned as described previously [32]. In brief, root tips were vacuum infiltrated with a fixative (1% paraformaldehyde, 5% DMSO, 0.1 M L-lysine, and 10 mM sodium-m-periodate in 20 mM phosphate buffer (PB) pH 7.2 for eGFP and pH 8.0 for mNeonGreen), fixed for 1 h at RT and rinsed with 20 mM PB pH 8.0. The root tips were sectioned with a vibrating-blade microtome, as described previously [31]. Cell walls were counterstained for 1 h with a fresh 0.1% solution of SCRI Renaissance Stain 2200 (SR2200, Renaissance Chemicals, Selby, UK) [92] in degassed ddH_2_O (pH 8.0), omitting subsequent rinsing. Longitudinal or cross sections (65 µm) of root tips or segments were mounted in consecutive order onto microscope slides in modified clearing reagent ClearSee [76] under coverslips. The components of ClearSee (10% xylitol (*w*/*v*), 15% (*w*/*v*) sodium deoxycholate, and 25% (*w*/*v*) urea) [93] were mixed in 20 mM PB (pH 8.0) and supplemented with 1.37% (*w*/*v*) L-lysine monohydrochloride (Sigma-Aldrich).

All microscopy procedures and the preparation of Maximum Intensity Projections were performed as described previously [31,32,76]. The examination and imaging of fluorescent protein patterns were performed under an LSM 780 or LSM 980 upright confocal laser scanning microscope (Carl Zeiss) equipped with a Plan-Apochromat 20×/0.8 numerical aperture DICII objective or a Plan-Apochromat 40×/1.3 numerical aperture DICIII oil immersion objective (Carl Zeiss). Samples were imaged with a 488 nm excitation laser line and an emission spectrum of 490–525 nm for mNeonGreen, with a 561 nm excitation laser line and an emission spectrum of 569–621 nm for mRuby3. For SR2200-stained cell walls or DAPI-stained nuclei, the 405 nm excitation laser line and an emission spectrum of 412–464 nm were used. ZEN 2.3pro or ZEN 3.5pro software (Carl Zeiss) was used for image processing. A multitrack (line-by-line) scan mode was applied for 405 nm and 561 nm channels. To eliminate the autofluorescence and separation of mNeonGreen fluorescence by 488 nm excitation laser line, a Linear Spectral Unmixing Algorithm or Emission Fingerprinting with Lambda stacks in ZEN was used [76,94]. The distance between the initial cell and the first cell in a file labeled with nuclear-localized mNeonGreen was measured using ZEN 2.3pro software after acquisition.

### 4.8. Statistical Analyses

Plots for RT-qPCR and treatment assays were prepared using the R programming language v. 4.0.2 [95] and RStudio software v. 1.3.1093 (posit.co, accessed on 1 May 2023). Code for the boxplot and the stripchart function from the base R package was used. The statistical analysis of the data was performed with Wilcoxon’s test from the base R package. Differences with *p*-values of <0.05 or <0.01 were considered statistically significant. The statistical analysis of *CsRALF34* expression in different plant organs was interpreted using the compact letter display methodology to clarify the presentation of results in the figure; the same letters indicate statistically indistinguishable variants. The RT-qPCR analysis of the relative expression levels of *RALF34* under auxin treatment was performed with five biological replicates, while four replicates were used for ACC/AIB treatment. For Cs*RALF34* expression in different cucumber organs, this analysis was performed using three biological replicates, with two independent RNA extractions (technical replicates) for each of them. At least 18 roots were used for each reporter gene assay.

## 5. Conclusions and Perspectives

This study shows many similarities in the expression of *CsRALF34* and *AtRALF34* during lateral root formation. However, since the *CsRALF34* product does not accumulate in the apoplast of cells involved in the initiation and early stages of lateral root primordia development, our results do not support the role of RALF34–THE1 signaling in the regulation of lateral root initiation. At this point, it is unclear why *CsRALF34* expression is induced only in the cucumber xylem and lateral root primordia cells.

Further studies of the RALF34 modulated signaling pathway, including identification of all participants of its receptor complex, are required to understand its role in root development. Comprehensive metabolomics and proteomics studies using overexpression or knockout of *RALF34* will help to shed light on the role of RALF34–THE1 in the modification of cortex cell walls in the basal part of the root meristem and how the perception of RALF34 is translated into changes in cell wall differentiation [96,97].

## Figures and Tables

**Table 1:** Names and functions of binary vectors used.

Name of Vector	Fusion Construct	Vector Purpose
pKGW-RR-MGW-*pCsRALF34::mNeonGreen-H2B*	*pCsRALF34::mNeonGreen-H2B*	*CsRALF34* gene expression analysis
pKGW-RR-MGW-*pCsRALF34::CsRALF34-mNeonGreen*	*pCsRALF34::CsRALF34-mNeonGreen*	CsRALF34 protein distribution analysis, *CsRALF34* fused to *mNeonGreen* without linker
pKGW-RR-MGW-*pCsRALF34::CsRALF34-linker-mNeonGreen*	*pCsRALF34::CsRALF34-linker-* *mNeonGreen*	CsRALF34 protein distribution analysis, *CsRALF34* fused to *mNeonGreen* via linker
pKGW-DR-MGW	*DR5::mRuby-H2B* in backbone	auxin response maxima via *DR5* reporter with nuclear localization
pKGW-DR-MGW- *pCsRALF34::CsRALF34-mNeonGreen*	*pCsRALF34::CsRALF34-mNeonGreen,* *DR5::mRuby-H2B*	CsRALF34 protein distribution analysis (*CsRALF34* fused to *mNeonGreen* without linker) in combination with *DR5*-reported auxin response maxima
pKGW-RR-MGW- *pCsTHESEUS1::mNeonGreen-H2B*	*pCsTHESEUS1::mNeonGreen-H2B*	*CsTHESEUS1* gene expression analysis

## Data Availability

All relevant data are included in this paper and its Supplementary Materials. The plasmid pKGW-DR-MGW will be available at Addgene (www.addgene.org, accessed on 1 May 2023).

## Supplementary Materials

The following supporting information can be downloaded at: https://www.mdpi.com/article/10.3390/ijms24098440/s1. References [98,99,100,101,102,103,104,105,106,107,108,109,110,111,112,113,114,115,116] are cited in the Supplementary Materials.

## Abbreviations

ACC, 1-aminocyclopropane-1-carboxylic acid; AIB, 2-aminoisobutyric acid; RT-qPCR, reverse transcription—quantitative polymerase chain reaction; RALF34, Rapid Alkalinization Factor 34.
