# Supplementary material for: Lateral Root Initiation in Cucumber (Cucumis sativus): What Does the Expression Pattern of Rapid Alkalinization Factor 34 (RALF34) Tell Us?
Source: Int J Mol Sci. 2023 May 8;24(9):8440. doi: 10.3390/ijms24098440 (PMC10179419; doi:10.3390/ijms24098440)
Supplement: Supplementary file 1 [file ijms-24-08440-s001.zip › Kiryushkin et al - RALF34 Supplementary Materials.pdf]

Supplementary Materials

Lateral Root Initiation in Cucumber (*Cucumis sativus*): What Does the Expression Pattern of *Rapid Alkalinization Factor 34 (RALF34)* Tell Us?

Alexey S. Kiryushkin, Elena L. Ilina, Elizaveta D. Guseva, Katharina Pawlowski and Kirill N. Demchenko

The following Supplementary Materials are available for this article:

Figure S1: Alignment and sequence logo of the RALF34 protein precursors across the Cucurbitales order compared with Arabidopsis;

Figure S2: Expression of the *RALF* genes in different cucumber organs;

Figure S3: Localization of RALF34 fusion protein in *Cucumis sativus* root tips;

Figure S4: Map of pKGW-DR-MGW binary vector;

Table S1: Information about Cucurbitales RALF34 sequences used in alignment for sequence logo building (Excel file).

Table S2: Construction of binary vectors; Table S3: Construction of entry and intermediate vectors;

Table S4: List of primers used for amplification of promoters/coding regions in this study;

Table S5: Combination of primers, used for different cloning steps;

Table S6: List of RT-qPCR primers used in this study;

Video S1: 3D movie of initiating lateral root primordium in *Cucumis sativus* root (*DR5::mRuby-H2B*). (AVI file).

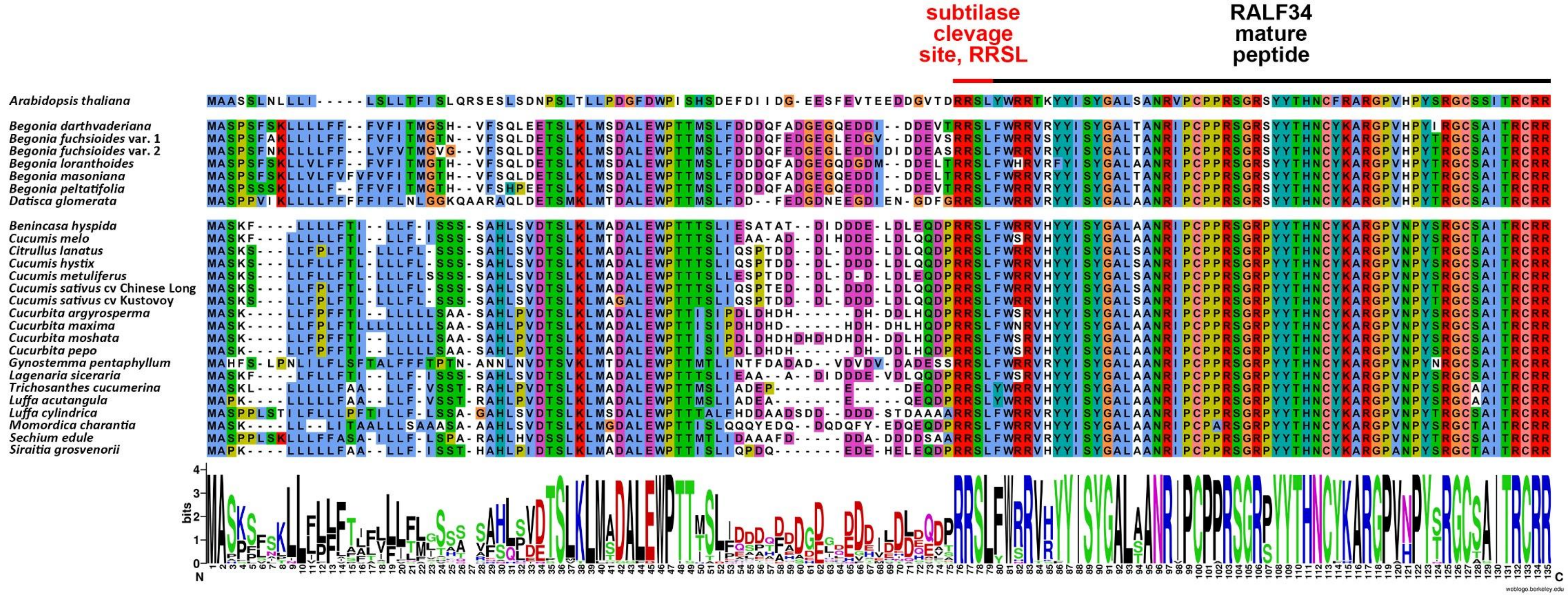

Figure S1. Alignment and sequence logo of RALF34 protein precursors within the Cucurbitales order compared with Arabidopsis. The RRSL subtilase cleavage site (red bold text) and the RALF34 mature peptide sequence (black bold text) are designated above the alignment. Size of letters in the sequence logo below the alignment is directly proportional to the amino acid similarity of the analyzed RALF34 proteins.

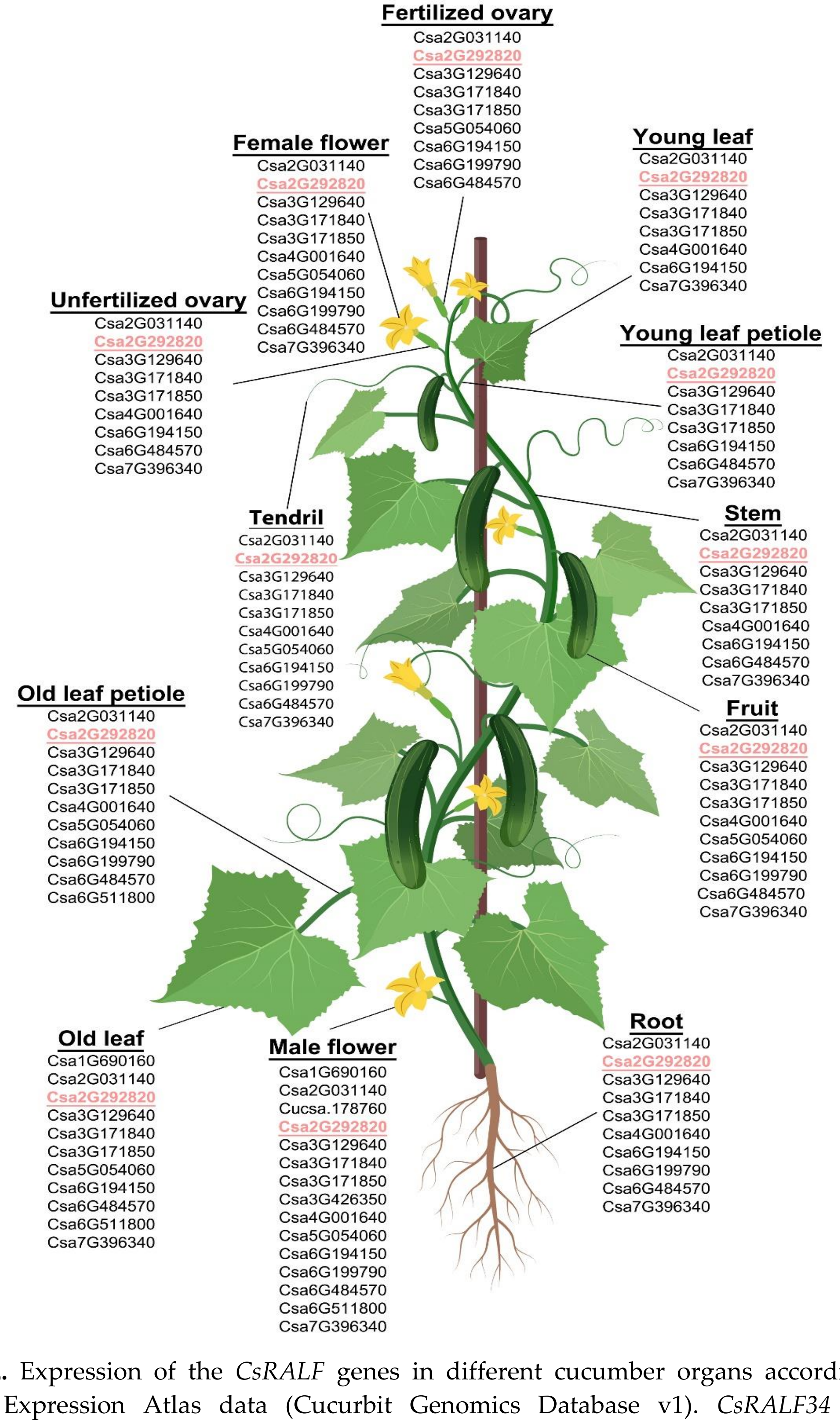

Figure S2. Expression of the *CsRALF* genes in different cucumber organs according to the Cucurbit Expression Atlas data (Cucurbit Genomics Database v1). *CsRALF34* gene (ID Csa2G292820) highlighted in light red is expressed in root, stem, old and young leaves including petioles, tendrils, male and female flowers, unfertilized and fertilized ovaries and in fruits. Figure was created with BioRender (https://biorender.com/).

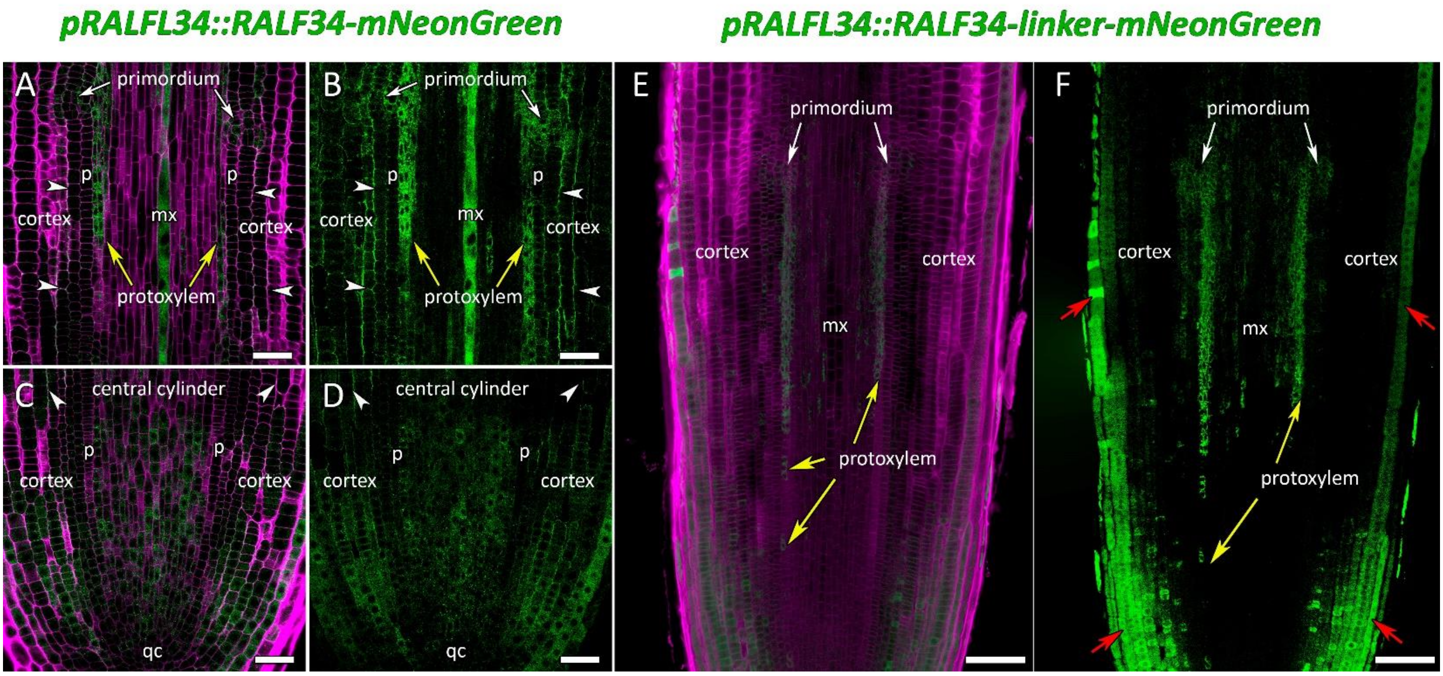

Figure S3. Localization of RALF34 fusion protein in *Cucumis sativus* root tips. Confocal laser scanning microscopy of longitudinal vibratome sections. (**A–D**) Fusion construct without linker (*pCsRALF34::CsRALF34-mNeonGreen*). (**A,B**) Basal part of the root apical meristem. Localization of CsRALF34 in the xylem and lateral root primordia and accumulation of fusion protein in the apoplast and along the cell walls of root cortex cells (white arrowheads). (**C,D**) Distal part of the root apical meristem. RALF34 fusion protein was detected only in the cytoplasm of cells. (**E,F**) Fusion construct with linker (*pCsRALF34::CsRALF34-linker-mNeonGreen*). No accumulation of fusion protein in the apoplast and along the cell walls of root cortex cells can be detected. Note the strong autofluorescence in the root cap cells (red arrows). Green channel – fluorescence of mNeonGreen (and autofluorescence); magenta channel – SR2200-stained cell walls. mx – metaxylem, p – pericycle; qc – quiescent centre. Scale bars: 50 μm in (**A–D**); 100 μm in (**E,F**).

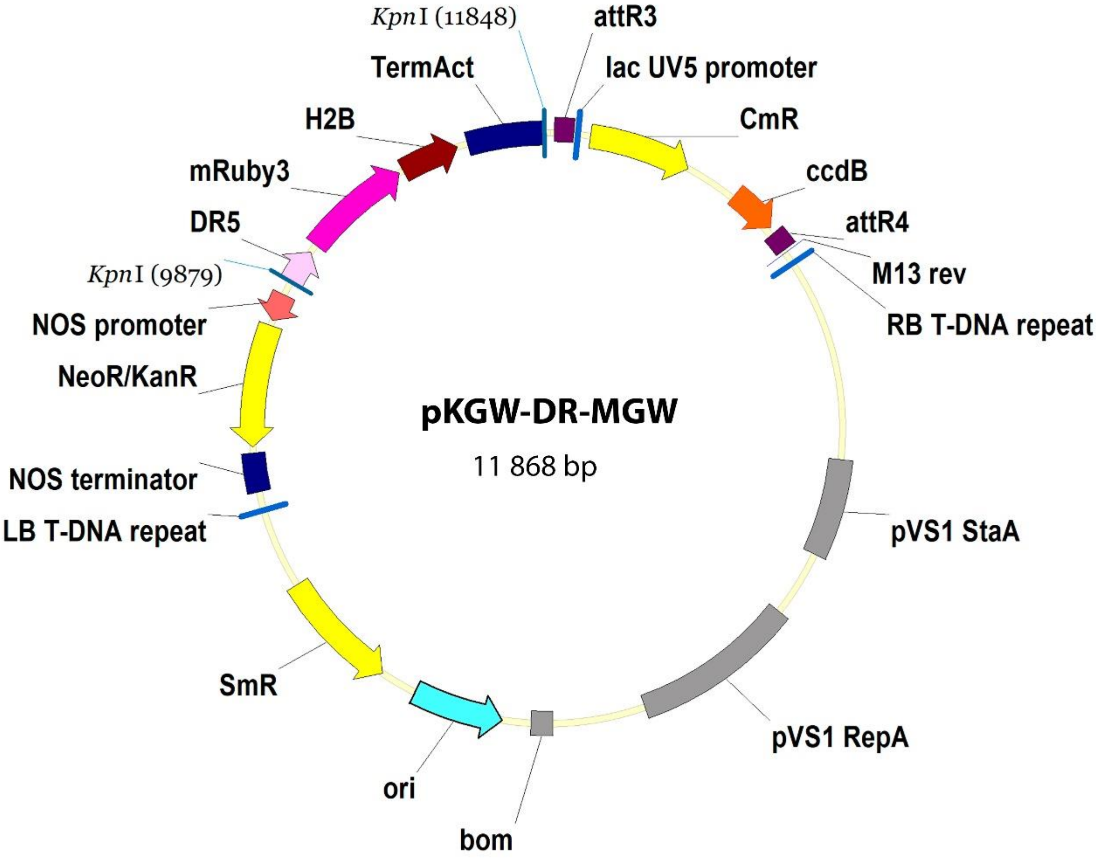

Figure S4. Map of the binary vector pKGW-DR-MGW.

Table S1. Information about Cucurbitales RALF34 sequences used in alignment for sequence logo building.

See:

Table S1. Information about Cucurbitales RALF34 sequences used in alignment for sequence logo building.xlsx

Table S2. Construction of binary vectors

Binary Vector	Destination vector	Promoter in entry vector	Reporter/insert in entry vector	Terminator in entry vector
**pKGW-RR-MGW-pCsRALF34::mNeonGreen-H2B**	pKGW-RR-MGW	pCsRALF34-pENTRattL4attR1_BSAI	mNeonGreen-H2B-pUC18-entry8	pENTRattR2attL3-TermAct
**pKGW-RR-MGW-pCsRALF34::CsRALF34-linker-mNeonGreen**	pKGW-RR-MGW	pCsRALF34-pENTRattL4attR1_BSAI	CsRALF34-linker-mNeonGreen-pUC18-entry8	pENTRattR2attL3-TermAct
**pKGW-RR-MGW-pCsRALF34::CsRALF34-mNeonGreen**	pKGW-RR-MGW	pCsRALF34-pENTRattL4attR1_BSAI	CsRALF34-mNeonGreen-pUC18-entry8	pENTRattR2attL3-TermAct
**pKGW-DR-MGW-pCsRALF34::CsRALF34-mNeonGreen**	pKGW-DR-MGW	pCsRALF34-pENTRattL4attR1_BSAI	CsRALF34-linker-mNeonGreen-pUC18-entry8	pENTRattR2attL3-T35S
**pKGW-RR-MGW-pCsTHESEUS1::mNeonGreen-H2B**	pKGW-RR-MGW	pCsTHESEUS1-pENTRattL4attR1_BSAI	mNeonGreen-H2B-pUC18-entry8	pENTRattR2attL3-TermAct
**pKGW-RR-MGW-DR5::mRuby3-H2B**	pKGW-RR-MGW	DR5-pENTRattL4attR1	mRuby3-H2B	pENTRattR2attL3-TermAct

Table S3. Construction of entry and intermediate vectors

Name of insert	Template	Source of template	Name of vector used/application	Source of vector used	Resulting entry/intermediate vector
**DR5**	DR5-pJET1.2	[1]	pDONR P4-P1R	Thermo Fisher Scientific	DR5-pENTRattL4attR1
**mRuby3-H2B**	Addgene plasmid #74258	a gift from Michael Lin	pUC18-entry8	[2]	mRuby3-H2B - pUC18-entry8
**pCsRALF34**	cucumber genomic DNA	current study	pENTRattL4attR1_BSAI	Wageningen University, Netherlands	pCsRALF34-pENTRattL4attR1_BSAI
**CsTHESEUS1 (promoter and coding region)**	cucumber genomic DNA	current study	pJET1.2	Thermo Fisher Scientific	CsTHESEUS1-pJET1.2
**pCsTHESEUS1**	CsTHESEUS1-pJET1.2	current study	pENTRattL4attR1_BSAI	Wageningen University, Netherlands	pCsTHESEUS1-pENTRattL4attR1_BSAI
**CsRALF34 (coding sequence)**	cucumber genomic DNA	current study	pJET1.2	Thermo Fisher Scientific	CsRALF34-pJET1.2
**CsRALF34-linker-mNeonGreen**	CsRALF34-pJET1.2/ Allele Biotechnology plasmid #H2B-213	current study/[3]	pUC18-entry8	[2]	CsRALF34-linker-mNeonGreen - pUC18-entry8

**CsRALF34-mNeonGreen**	CsRALF34-pJET1.2/ Allele Biotechnology plasmid #H2B-213	current study/[3]	pUC18-entry8	[2]	CsRALF34-mNeonGreen -pUC18-entry8

Table S4. List of primers used for amplification of promoters/coding regions in this study

Name	Restriction enzyme/ att site	Sequence 5`-3`
DR5_FOR5	attB4	GGGG**ACAACTTTGTATAGAAAAGTTG**GGTATCGCAGCCCCCTTTTGTCTCC
DR5_REV3	attB1r	GGGG**ACTGCTTTTTTGTACAAACTTG**TGTTGTTTGTTGTTTGTTGTTGTTGGTAATTGTTG
DR5_FOR4	*Kpn*I	AAA**GGTACC**GGTATCGCAGCCCCCTTTTGTCTCC
TermAct_REV	*Kpn*I	AAA**GGTACC**CTCAAGCGAAATGGTGCGATCT
T35S_REV	*Kpn*I	AAA**GGTACC**tcactggattttggttttaggaatt
pCsRALF34_FOR	*Sma*I	A**CCCGGG**CAAATTATAGGGATGAGCATTGGTTCG
pCsRALF34_REV	*Sma*I	A**CCCGGG**GGGGTTTAGAGAGAGAGAGATGAATGTCAC
pCsTHESEUS1_FOR	*Xho*I	AA**CTCGAG**AGAAATAGGATGACTTGAACATGAACCTCC
pCsTHESEUS1_REV	*Kpn*I	AAA**GGTACC**AACTCCATTGAAGAACACAAGAATCTAAAC
CDS_CsTHESEUS1_REV	-	ACCCGGGAACTCCATTGAAGAACACAAGAATCTAAAC
CDS_CsRALF34_FOR	-	TTGAAAACCGACACTAAAAAACAAGAA
CDS_CsRALF34_REV	-	ATATAAAATAAGGAAATCCCCAAAACTACA
CDS_CsRALF34_over_FOR	*Kpn*I	AAA**GGTACC**ATGGCTTCCAAATCCCTCCTCTT
CDS_CsRALF34_over_REV	-	CACTCGCTGCCGCCGCATGGTGAGCAAGGGCGAGGAGGATAACATGGCCTCTCTCC
CDS_CsRALF34_over_linker_REV	-	CACTCGCTGCCGCCGCGATCCACCGGTCGCCACCATGGTGAGCAAGGGCGAGGAGG
mNeonGreen_FOR	-	ATGGTGAGCAAGGGCGAGGA
mNeonGreen_REV	*Not*I	AA**GCGGCCGC**TTACTTGTACAGCTCGTCCATGCC
CDS CsRALF34_REV1	*Not*I	AA**GCGGCCGC**TCAGCGGCGGCAGCGA
mRuby3_FOR	*Bam*HI	AA**GGATCC**ATGGTGTCTAAGGGCGAAGAGC
H2B_REV	*Not*I	AA**GCGGCCGC**TTACTTAGCGCTGGTGTACTTGG

Restriction enzyme/att sites in adaptors are underlined and given in **BOLD** style.

Table S5. Combination of primers, used for different cloning steps

Combination of primers	Application
DR5 FOR5/DR5 REV3	PCR amplification of DR5 product with attB4/attB1r adaptors for subsequent cloning into pDONR P4-P1R by BP-clonase reaction
mRuby3 FOR/H2B REV	PCR amplification of mRuby3-H2B product for subsequent *Bam*HI/*Not*I cloning into pUC18-entry8
DR5_FOR4/TermAct REV	DR5::mRuby3-H2B-TermAct insert verification by PCR in pKGW-RR-MGW vector
DR5_FOR4/TermAct REV	PCR amplification of DR5::mRuby3-H2B-TermAct cassette for subsequent *Kpn*I cloning into pKGW-MGW backbone
DR5_FOR4/TermAct REV	DR5::mRuby3-H2B-TermAct insert verification by PCR in pKGW-DR-MGW vector backbone
pCsRALF34 FOR/pCsRALF34 REV	PCR amplification of pCsRALF34 promoter using cucumber genomic DNA as a template
pCsRALF34 FOR/ TermAct REV	pCsRALF34::mNeonGreen-H2B-TermAct, pCsRALF34::CsRALF34-linker-mNeonGreen-TermAct, pCsRALF34::CsRALF34-mNeonGreen-TermAct insert verification by PCR in pKGW-RR-MGW vector
pCsTHESEUS1 FOR/CDS CsTHESEUS1 REV	PCR amplification of *CsTHESEUS1* 6000 bp-fragment containing promoter region and coding sequence for subsequent cloning to pJET1.2
pCsTHESEUS1 FOR/ pCsTHESEUS1 REV	pCsTHESEUS1 insert verification by PCR in pJET1.2 and pENTRattL4attR1_BSAI
pCsTHESEUS1 FOR/ pCsTHESEUS1 REV	PCR amplification of p*CsTHESEUS1* promoter for subsequent *Xho*I-*Kpn*I cloning into pENTRattL4attR1_BSAI
CDS CsRALF34 FOR/ CDS CsRALF34 REV	PCR amplification of *CsRALF34* coding sequence using cucumber genomic DNA as a template for subsequent cloning to pJET1.2
CDS CsRALF34 over FOR/CDS CsRALF34 over linker REV	PCR amplification of *CsRALF34* coding sequence fused to linker for further usage as a template in overlap extension PCR
CDS CsRALF34 over FOR/CDS CsRALF34 over REV	PCR amplification of *CsRALF34* coding sequence (without linker) for further usage as a template in overlap extension PCR
mNeonGreen FOR/ mNeonGreen REV	PCR amplification of mNeonGreen coding sequence for further usage as a template in overlap extension PCR
CDS CsRALF34 over FOR/ mNeonGreen REV	Amplification of final PCR product in overlap extension PCR using *CsRALF34* (with or without linker) and *mNeonGreen* fragments obtained during previuos steps and subsequent *Kpn*I-*Not*I cloning to pUC18-entry8

Table S6. List of RT-qPCR primers used in this study.

Name	Sequence 5′-3′	Amplicon size, bp	Application
CsRALFL34 FOR1	CGTAGGGAAGGAGTGAAGAGGTGG	160	Expression of *CsRALFL34* in response to auxin and ethylene
CsRALFL34 REV1	TGGATGAGGGAAGTGGTGGTGG		
CsRALFL34 FOR2	CTCCAATCACACTCTCACTCTCTTCTCCT	75	Expression of *CsRALFL34* in different organs of cucumber
CsRALFL34 REV3	AGAGGAGGGATTTGGAAGCCATTG		
CsEF1a FOR	ATGGGTAAGGAGAAGGTTCACATTAACATT	241	Reference gene
CsEF1a REV	CGAACTTCCACAAAGCAATATCAATT		

Video S1
See:
Video S1 DR5-mRuby-H2B in Cucumis sativus.avi

Supplementary references

1. Ilina, E.L.; Logachov, A.A.; Laplaze, L.; Demchenko, N.P.; Pawlowski, K.; Demchenko, K.N. Composite *Cucurbita pepo* plants with transgenic roots as a tool to study root development. *Ann. Bot.* **2012**, *110*, 479-489, doi:10.1093/aob/mcs086.
2. Hornung, E.; Krueger, C.; Pernstich, C.; Gipmans, M.; Porzel, A.; Feussner, I. Production of (10*E*,12*Z*)-conjugated linoleic acid in yeast and tobacco seeds. *Biochim. Biophys. Acta - Mol. Cell Biol. Lipids* **2005**, *1738*, 105-114, doi:10.1016/j.bbalip.2005.11.004.
3. Shaner, N.C.; Lambert, G.G.; Chammas, A.; Ni, Y.; Cranfill, P.J.; Baird, M.A.; Sell, B.R.; Allen, J.R.; Day, R.N.; Israelsson, M., et al. A bright monomeric green fluorescent protein derived from *Branchiostoma lanceolatum*. *Nat. Methods* **2013**, *10*, 407-409, doi:10.1038/nmeth.2413.
